# Adaptive structural changes in the motor cortex and white matter in Parkinson’s disease

**DOI:** 10.1007/s00401-022-02488-3

**Published:** 2022-09-02

**Authors:** YuHong Fu, Liche Zhou, Hongyun Li, Jen-Hsiang T. Hsiao, Binyin Li, Onur Tanglay, Andrew D. Auwyang, Elinor Wang, Jieyao Feng, Woojin S. Kim, Jun Liu, Glenda M. Halliday

**Affiliations:** 1 Brain and Mind Centre & Faculty of Medicine and Health School of Medical Sciences, The University of Sydney, Sydney, NSW 2050 Australia; 2 Department of Neurology and Institute of Neurology, Ruijin Hospital, Shanghai Jiao Tong University School of Medicine, Shanghai, 200025 China; 3 Neuroscience Research Australia & Faculty of Medicine School of Medical Sciences, University of New South Wales, Sydney, NSW 2052 Australia

**Keywords:** α-Synuclein, Axon, Diffusion tensor imaging, Myelin, Oligodendrocyte, Parkinson’s disease

## Abstract

Parkinson’s disease (PD) is a movement disorder characterized by the early loss of nigrostriatal dopaminergic pathways producing significant network changes impacting motor coordination. Recently three motor stages of PD have been proposed (a silent period when nigrostriatal loss begins, a prodromal motor period with subtle focal manifestations, and clinical PD) with evidence that motor cortex abnormalities occur to produce clinical PD[8]. We directly assess structural changes in the primary motor cortex and corticospinal tract using parallel analyses of longitudinal clinical and cross-sectional pathological cohorts thought to represent different stages of PD. 18F-FP-CIT positron emission tomography and subtle motor features identified patients with idiopathic rapid-eye-movement sleep behaviour disorder (*n* = 8) that developed prodromal motor signs of PD. Longitudinal diffusion tensor imaging before and after the development of prodromal motor PD showed higher fractional anisotropy in motor cortex and corticospinal tract compared to controls, indicating adaptive structural changes in motor networks in concert with nigrostriatal dopamine loss. Histological analyses of the white matter underlying the motor cortex showed progressive disorientation of axons with segmental replacement of neurofilaments with α-synuclein, enlargement of myelinating oligodendrocytes and increased density of their precursors. There was no loss of neurons in the motor cortex in early or late pathologically confirmed motor PD compared to controls, although there were early cortical increases in neuronal neurofilament light chain and myelin proteins in association with α-synuclein accumulation. Our results collectively provide evidence of a direct impact of PD on primary motor cortex and its output pathways that begins in the prodromal motor stage of PD with structural changes confirmed in early PD. These adaptive structural changes become considerable as the disease advances potentially contributing to motor PD.

**Supplementary Information:**

The online version contains supplementary material available at 10.1007/s00401-022-02488-3.

## Introduction

Parkinson’s disease (PD) is a progressive neurodegenerative disorder characterised by the motor symptoms of bradykinesia, rigidity and resting tremor that are known to be caused by the loss of dopaminergic neurons in the substantia nigra pars compacta [48]. The initial evolution of PD has recently been divided into three different periods based on motor phenotype and the severity of nigrostriatal degeneration [8]: (1) a silent motor period when nigrostriatal degeneration begins but functional compensation occurs; (2) a prodromal motor period when subtle breakthrough focal motor manifestations occur due to abnormalities in the motor network; and (3) clinically progressive PD with the diagnostic motor symptoms. Significant neuronal loss in the silent period is relatively confined to the nigrostriatal system [8, 27], but compensatory and pathophysiological changes in the prodromal motor period extend to the larger motor network [8]. Recent data show that the evolution of motor PD occurs in concert with changes in the motor cortex [8]. In particular, motor cortex excitability is already altered in the prodromal PD period prior to diagnosis [30, 32], but pyramidal tract impairment is considered a “red flag” against a diagnosis of PD [48], suggesting functional rather than significant structural changes impact this brain region in PD.

In addition to the loss of midbrain dopaminergic neurons, the other neuropathological hallmark of PD is the abnormal neuronal aggregations of α-synuclein (αSyn) in Lewy pathology which propagates through different brain regions in a standard pattern [9]. Aggregated αSyn disrupts the microtubule network and trafficking [34], and the actin cytoskeleton [44], potentially impairing neuronal function and survival. Pathological Lewy inclusions have been linked to the non-motor symptoms of PD [8, 16]. Of note, the motor cortex is largely spared Lewy pathology until the last disease stage when it is mildly affected [9], a feature which has led to its exclusion from many studies. Our previous stereological analysis of neurons in the primary motor cortex in early PD cases revealed no significant loss of pyramidal neurons (neurofilament-containing or those with other intermediate filaments) or interneurons compared with controls [24]. We also confirmed that primary motor cortex has limited Lewy pathology at this early stage [24]. Neurofilaments are composed of a triplet of proteins by weight (heavy (NFH), medium (NFM) or light (NFL) chain), with NFL identified as a peripheral biomarker of axonal injury in different neurological disorders [29, 53]. In de novo PD patients, baseline serum NFL levels predict the rate of motor decline, the accumulation of additional motor features, and the progression of dopamine transporter loss in early PD [67]. Higher cerebrospinal fluid (CSF) levels of NFL in early PD associate with greater severity of all cardinal PD motor symptoms, greater loss of striatal dopamine transporter uptake, and lower fractional anisotropy (FA) in diffusion tensor imaging (DTI) of several axonal tracts [6]. The presence of some Lewy pathology at early disease stages suggests that there are subtle structural changes occurring in primary motor cortex in PD that warrant further assessment, particularly if these changes are those involved in the evolution of motor PD [8].

To analyse the different stages of motor PD, we conducted parallel studies on the microstructure of the motor cortex and corticospinal tract in different cohorts. We first assessed these structures using DTI in longitudinally studied patients with idiopathic rapid-eye-movement (REM) sleep behaviour disorder (iRBD) who converted to prodromal PD (*n* = 8) and age-matched controls (*n* = 20). iRBD itself is considered a prodromal stage of α-synucleinopathy [49]. We next examined these structures in post-mortem confirmed motor PD cases with either early or end stage Lewy body disease [9].

## Materials and methods

### Assessment of motor cortex and tracts longitudinally in a clinical cohort

#### Silent and prodromal clinical participants

Participants with iRBD were recruited to this longitudinal study through Ruijin Hospital between 2017 and 2019. The study was approved by the Ethics Committee at Ruijin Hospital affiliated with School of Medicine, Shanghai Jiao Tong University (2018/101). All participants met the following criteria: (a) age at baseline of between 56 and 73 years old, (b) no sign of dementia using both the Mini-Mental State Examination (MMSE, Chinese version cut-off point 24/25 for participants with more than 6 years of education [14]) and Montreal Cognitive Assessment (MoCA) [42], (c) no history of intracranial surgery or traumatic brain injury, (d) no psychiatric disorders, (e) no alcohol use disorder, and (f) no history of other neurological disorders. These participants had two visits (baseline and the follow-up) with an interval of around 2 years for diffusion magnetic resonance imaging (dMRI) scans and assessments including the Scale for Outcomes in PD-Autonomic (SCOPA-AUT [66]) and iRBD screening questionnaire (RBDSQ) [59]. Healthy controls (*n* = 20) and the selected iRBD patients at the first visit (*n* = 8) neither complained of any motor symptom nor presented with parkinsonism or another neurological illness. The absence of motor parkinsonism or other neurological signs was confirmed by two experienced neurologists. iRBD patients had typical dream enactment and were diagnosed based on clinical assessment and video-polysomnographic evidence (E-Series electroencephalography (EEG) and polysomnography (PSG) Recording System, Compumedics Ltd, Melbourne, Australia) according to the 3rd edition of the International Classification of Sleep Disorders [54]. At the second visit, these selected iRBD cases had developed either prodromal motor PD identified by two movement disorders specialists and decreased basal ganglia dopamine transporter (DAT) using 18F-N-(3-fluoropropyl)-2β-carbon ethoxy-3β-(4-iodophenyl) nortropane (18F-FP-CIT) hybrid positron emission tomography (PET)/ magnetic resonance imaging (MRI), or had silent PD with only decreased basal ganglia DAT and no symptoms. The diagnostic threshold for motor PD [48] using the Movement Disorder Society Unified Parkinson’s Disease Rating Scale (MDS-UPDRS) [20] was not met in the iRBD patients, consistent with preclinical stages of PD. Demographics of all clinical participants are listed in Table 1. There were no significant differences in age, sex, follow-up years, MMSE or MoCA scores between the groups using Mann–Whitney and Chi-squared tests (see *p* values in Table 1).

**Table 1:** Demographics of the cohorts studied

	iRBD (*n* = 8)		Healthy control (*n* = 20)		*p* value	
	Baseline	Follow-up	Baseline	Follow-up	Baseline	Follow-up
Clinical participants						
Age (years)	64.1 ± 7.2	66.6 ± 7.2	62.6 ± 6.0	65.2 ± 6.0	0.61	0.63
Gender (M/F)	5/3		7/13		0.18	
Follow-up time (years)	2.2 ± 0.5		2.4 ± 0.5		0.34	
Time since medical diagnosis (years)	2.8 ± 0.5		–		–	
Time since onset of symptoms (years)	7.9 ± 5.0		–		–	
MDS-UPDRS III score [21]	0.4 ± 1.1	4.9 ± 4.5	–		0.002	
RBDSQ [62]	8.8 ± 2.3	9.6 ± 1.8	0.7 ± 0.7	0.8 ± 0.9	< 0.001	< 0.001
cMMSE [14]	28.8 ± 1.2	28.5 ± 1.2	28.5 ± 1.3	28.7 ± 1.0	0.73	0.75
MoCA [45]	–	26.1 ± 3.1	–	27.1 ± 1.8	–	0.64
SCOPA-AUT [69]	10.1 ± 4.3	12.6 ± 5.5	3.0 ± 2.4	4.2 ± 4.4	< 0.001	< 0.001

Group	Gender (M/F)	Age at death (years)	Disease duration (years)	Post-mortem delay (hours)	Braak Lewy stage (/6)
Post-mortem cohort					
IHC/IF					
Control	5/5	77.8 ± 10.0	–	15.5 ± 6.4	–
PD-eLP	5/5	76.5 ± 5.9	11.6 ± 2.8	14.3 ± 7.6	4.0 ± 0.0
PD-lLP	6/4	79.9 ± 4.4	14.3 ± 7.8	9.5 ± 9.5	6.0 ± 0.0
WB-GM					
Control	3/5	81.4 ± 4.3	–	22.5 ± 9.2	–
PD	7/5	77.8 ± 5.5	12.8 ± 5.3	18.3 ± 12.6	4.8 ± 0.9
WB-WM					
Control	7/3	78.9 ± 10.5	–	23.9 ± 8. 3	–
PD	6/2	80.3 ± 3.4	13.5 ± 6.3	20.4 ± 11.5	5.6 ± 0.4

Values are presented as mean ± standard deviation. Comparison was done with Mann–Whitney test, except for gender difference used chi-square test. *PD* Parkinson’s disease, *iRBD* idiopathic rapid eye movement sleep behaviour disorder patients that converted to silent and/or prodromal motor PD, *MDS-UPDRS III* Movement Disorder Society Unified PD Rating Scale part III, *RBDSQ* REM sleep behaviour disorder screening questionnaire, *cMMSE* Chinese version of the Mini-Mental State Examination, *MoCA* Montreal Cognitive Assessment, *SCOPA-AUT* Scale for Outcomes in PD-Autonomic, *WB* western blot, *GM* grey matter, *WM* white matter, *IHC/IF* immunohistochemistry/immunofluorescence, *PD-eLP* PD cases with early Lewy pathology (Braak stage 4), *PD-lLP* PD cases with late Lewy pathology (Braak stage 6)

### MRI and PET acquisition

MRI data acquisition was performed using a Siemens Trio 3 T MRI scanner equipped with a 12-channel head coil at baseline. T1-weighted images were obtained using a 3D magnetization-prepared rapid acquisition gradient-echo (MPRAGE) sequence (176 axial slices, flip angle of 9°, 1 × 1 × 1 mm^3^ voxel size, echo time/repetition time/inversion time = 3.0/2300/1000 ms) for volumetric and registration purposes. DTI data were acquired using the following parameters: field of view (FOV) = 220 mm^2^, 42 slices, slice thickness of 3 mm, voxel size of 1.7 × 1.7 × 3.0 mm^3^, TE of 94 ms, TR of 6000 ms, b-values of 0 and 1000 s/mm^2^, and diffusion gradient directions of 30.

All 18F-FP-CIT hybrid PET/MRI examinations were performed on a Siemens Biograph mMR scanner (Siemens Healthcare, Erlangen, Germany) using an 8-channel phase-array head coil and mean dose of 3.7 MBq/kg body weight (supplied by Ruijin Hospital, School of Medicine, Shanghai Jiao Tong University). Static 18F-FP-CIT PET data were acquired in sinogram mode for 15 min using the following parameters: 128 slices per slab, gap 0.5 mm; matrix size 344 × 344, an iterative reconstruction based on ordered subset expectation maximization (OSEM) with 21 subsets, 4 iterations and post-filtered with an isotropic full-width half-maximum (FWHM) Gaussian kernel of 2 mm. Advanced PET attenuation correction was used with a unique 5-compartment model that includes bone. Follow-up dMRI imaging was performed simultaneously with PET data acquisition for all iRBD patients and solely applied to healthy controls.

### Image pre-processing

Baseline 4D dMRI data using UK biobank protocol (https://www.ukbiobank.ac.uk/) for Eddy currents and for head motion outlier-slices (individual slices in the 4D data) using the Eddy tool (available from http://fsl.fmrib.ox.ac.uk/fsl/fslwiki/EDDY) [2, 3]. Since full 3D gradient distortion correction (GDC) was not available on the scanner for echo-planar imaging, GDC was applied to images after eddy correction using available online tools (https://github.com/Washington-University/Pipelines). This correction requires a proprietary data file (.coeff or .grad) from Siemens which describes the gradient nonlinearities. After these corrections, the 4D *b* = 1000 shell (30 directions) was uploaded to the DTI fitting tool DTIFIT to generate FA. The DTI FA images were then processed with Tract-Based Spatial Statistics (TBSS), which aligned FA images onto a standard-space white matter (WM) skeleton with a typical threshold value of 0.2 for further comparison [15, 57].

### Structural assessment of post-mortem motor cortex and underlying white matter

#### Post-mortem motor PD and age-matched controls

Tissue samples from pathologically confirmed PD brains (*n* = 40) and age and post-mortem delay matched controls without neurological or neuropathological disease (*n* = 28) were obtained from the New South Wales (NSW) Brain Banks (demographics in Table 1, no significant difference in age, sex or post-mortem delay using Mann–Whitney tests and Chi-squared tests). The study was approved by the University of Sydney Human Research Ethics Committee (2019/491). All cases with PD were levodopa-responsive and fulfilled the UK Brain Bank Clinical Criteria for a diagnosis of PD [16] with no other neurodegenerative conditions. Controls by definition had no Lewy pathology, while PD cases were staged using the regional location of Lewy pathology [9] and had Braak stages of IV–VI. Formalin-fixed paraffin-embedded (FFPE) sections from controls, PD cases with early Lewy pathology (Braak stage IV), and PD cases with late Lewy pathology (Braak stage VI) (*n* = 10 cases per group) were used to examine the structural and cytoarchitecture changes in the primary motor cortex (superior precentral gyrus) and subcortical WM. This region of the primary motor cortex controls trunk and lower limb function [19]. Fresh-frozen tissue from the motor cortex (*n* = 8 controls and *n* = 12 PD cases) and subcortical WM (*n* = 10 controls and *n* = 8 PD cases) were used to extract sodium dodecyl sulphate (SDS)-soluble fractions for measuring the constituent levels of axonal, myelin, and oligodendrocyte proteins.

### Immunohistochemistry and immunofluorescence

FFPE sections of primary motor cortex were cut at 8 µm with a rotary microtome (Thermo/Microm, HM325). Sections were mounted on Series 2 adhesive microscope slides (Trajan Scientific Medical, AU). Before staining, sections were de-waxed and rehydrated with xylene and a series of ethanol. Each antibody was first tested with immunohistochemistry (IHC) to decide the optional heat-induced antigen retrieval (HIAR). This included sodium citrate buffer (pH 6.0) and additional formic acid treatment (70% concentration for 30 min) for αSyn staining. For IHC, sections were blocked with 5% normal horse serum, then incubated with primary antibodies for 48 h at 4 °C, followed by the secondary antibody ImmPRESS™- alkaline phosphatase (AP), horseradish peroxidase (HRP) anti-mouse, or rabbit IgG polymer detection kit (VectorLabs, see also supplement Table 1), developed with ImmPACT^®^ DAB EqV HRP substrate (VectorLabs, cat# SK-4103) or ImmPACT^®^ Vector^®^ red substrate AP (VectorLabs, cat# SK-5105) according to the company’s instructions. Sections were then counterstained with haematoxylin or cresyl violet and coverslipped.

Immunofluorescence (IF) co-labelling using combinations of primary antibodies (see Supplementary Table 1, online resource) was then optimised to allow three antigens to be detected simultaneously. HIAR was performed in a programmable antigen retrieval cooker (Aptum Bio Retriever 2100, Aptum Biologics Ltd, UK) at a peak temperature of ~ 121 °C, followed by gradual cooling for two hours. After HIAR, sections were immersed in phosphate buffered saline (PBS) with 0.1% sodium borohydride for 30 min on ice. After washing with PBS, sections were incubated with 100 mM glycine in PBS for 30 min; then elimination of lipofuscin autofluorescence was performed with 0.1% Sudan Black in 70% ethanol for 30 min. After treatment with blocking buffer (containing 2% normal serum and 1% bovine serum albumin (BSA) in PBS) for 1 h at room temperature, the slides were incubated with the cocktail of primary antibodies in blocking buffer at the appropriate dilutions for 48 h at 4 °C, followed by their corresponding Alexa Fluor 488/568/647 secondary antibodies (dilution 1:250, see Supplementary Table 1 for secondary antibodies, online resource) and 4′,6-diamidino-2-phenylindole (DAPI, Sigma cat# D9542, 1 mg/ml) for 2 h at room temperature. To further quench autofluorescence, the fluorophore-labelled slides were finally treated with 10 mM CuSO_4_ in 50 mM ammonium acetate buffer (pH 5.0) for 1 h before being mounted with mounting medium (DAKO, cat# S3023) and sealed with nail polish. Negative controls were performed for each batch of staining by omitting either the primary or secondary antibodies.

### Image capture, processing, and analysis

Stained IHC and IF sections were scanned using an Olympus VS120 Slide Scanner with consistent settings applied for the same parameter, including axonal coherency of NF-L labelled neuronal axons, and cell counting and size measurements.

Axonal coherency in the WM was assessed in IHC sections of NF-L. Digital images were adjusted for orientation and cropped for sampling the same sized regions of interest (ROI; 1050 × 700 μm^2^) using the VS-DESKTOP software (Olympus Soft Imaging Solutions GmbH, ver. 2.9 13753). The anisotropic properties of axons were characterised by their directional coherency coefficient, which was calculated by the ‘OrientationJ’ Java plugin for the ImageJ/Fiji software (ImageJ version 2.1.0/1.53c bundled with Java 8) [18, 56] as shown in the workflow for axonal orientation (Supplementary Fig. 1, online resource). ‘OrientationJ’ operates by evaluating the gradient structure tensor over Gaussian-shaped windows in the analysed image as described below.

$$J = \left[ {\begin{array}{*{20}c} {\left\langle {f_{x} ,f_{x} } \right\rangle_{w} } & {\left\langle {f_{x} ,f_{y} } \right\rangle_{w} } \\ {\left\langle {f_{x} , f_{y} } \right\rangle_{w} } & {\left\langle {f_{y} , f_{y} } \right\rangle_{w} } \\ \end{array} } \right]$$

where *J* is the structure tensor, *f*_*x*_ and *f*_*y*_ are the partial spatial derivatives of the image, and *w*(*x, y*) is the weighting function [18, 51]. The dominant orientation of axons in each ROI, and the extent to which the ROI aligns with the dominant orientation (i.e., coherency) can be estimated upon analysing its structure tensor [12, 51]. Mathematically, the coherency parameter is calculated by the following ratio between the largest and smallest eigenvalues of the structure tensor:

$$C = \frac{{\lambda_{{{\text{max}}}} - \lambda_{{{\text{min}}}} }}{{\lambda_{{{\text{max}}}} + \lambda_{{{\text{min}}}} }} = \frac{{\sqrt {\left( {\left\langle {f_{y} , f_{y} } \right\rangle_{w} - \left\langle {f_{x} ,f_{x} } \right\rangle_{w} } \right)^{2} + 4\left\langle {f_{x} , f_{y} } \right\rangle_{w} } }}{{\left\langle {f_{x} ,f_{x} } \right\rangle_{w} + \left\langle {f_{y} , f_{y} } \right\rangle_{w} }}$$

where *C* is the coherency, *λ*_max_ is the largest eigenvalue of the structure tensor, and *λ*_min_ is the smallest eigenvalue of the structure tensor. A coherency value of 1 suggests that the ROI has one dominant orientation, whereas a coherency value of 0 suggests that there is no dominant orientation, and that the ROI is isotropic [18, 50]. Principles behind Orientation J’s computations have been reported previously [18, 51].

The number and size of oligodendrocytes (OLGs) were assessed in IF sections labelled for tubulin polymerization promoting protein (TPPP)/P25α and DAPI. TPPP/P25α is expressed mainly in mature OLGs and it aligns the microtubule system during the process elongation prior to the onset of myelination [35]. For each case, two non-overlapping ROIs (980 × 650 μm^2^) were sampled from the superficial cortical grey matter (SGM), deep cortical grey matter (DGM), and WM, respectively. Parameters (size and circularity) for auto-quantification were determined by histograms of TPPP/P25α^+^ cells and DAPI^+^ nuclei from each brain region to include approximately 90% of the sampled cell population. The images were binarized for automatic thresholding, the size and number of TPPP/P25α^+^ OLGs, and the number of DAPI^+^ nuclei were quantified (ImageJ version 2.0.0-rc-69/1.52p). The percentage of TPPP/p25α^+^ OLGs as a proportion of DAPI^+^ nuclei was calculated for each ROI.

The morphology of cell types, pathology, axons and myelin were assessed in IF images captured using a confocal microscope (Nikon C2) with parameters (laser power, gain, and offset) set based on the negative control and single channel labelling. Sequential scanning starting from the longest wavelength was used to avoid signal bleaching between channels, and single labelled sections were compared with co-labelled sections to ensure no interfering effects (antibody competition or cross-reaction). Consistent image settings were applied to the sections stained with the same combination of antibodies. All post-acquisition brightness and contrast adjustments were equivalently applied to sections stained with the same sets of antibodies with Fiji (ImageJ version 2.1.0/1.53c). The mean grey value of NFL signals and the diameter of NFL-labelled axonal structure were measured with Fiji in 20 axons within a randomly selected ROI per case. Adjustment of brightness and contrast was applied for image presentation. The relationship between axons and myelin sheaths was assessed in images captured using high stimulated emission depletion microscopy (Leica TCS SP8 STED) and LASX software (version 3.7.4).

### Fractional protein extraction and western immunoblotting

Soluble and insoluble proteins were serially extracted from fresh-frozen brain tissue of primary motor cortex and subcortical WM (50 mg each) as previously described [71]. Tissue was homogenized with tungsten beads by TissueLyser (Qiagen, Hilden, Germany) in tris-buffered saline (TBS) homogenization buffer [(50 mM Tris, 125 mM NaCl, pH 7.4, 5 mM EDTA, 0.02% sodium azide containing protease inhibitor cocktail (Complete, EDTA-free; Roche 04,693,132,001)], centrifuged at 120,000 *g* for 2 h at 4 °C and the supernatant collected as the TBS-soluble fraction containing extracellular and cytosolic proteins. The pellet was re-suspended in TBS homogenization buffer containing 5% SDS, centrifuged at 100 000 g for 30 min at 25 °C, and the supernatant collected as the SDS-soluble fraction mostly containing membrane-associated proteins. Protein concentration of all fractions was quantified using the bicinchoninic acid (BCA) protein assay (Pierce BCA Protein Assay Kit; Thermo Scientific 23225) according to manufacturer instructions. Samples were stored at − 80 °C before measurement.

SDS-fractions were assessed with western immunoblotting (WB) for the levels of αSyn, neuronal cytoskeleton proteins, and OLGs/myelin proteins. Equal amounts of protein lysates were resolved with loading buffer (2% SDS, 20% glycerol, 2.5% bromophenol blue, 12.5 mM Tris–HCl, pH 6.8, 5% 2-mercaptoethanol) in 4–20% SDS-PAGE gradient criterion gels (Bio-Rad, USA), then transferred onto 0.2 μm nitrocellulose membranes. Membranes were visualised using ChemiDoc MP imaging system (Bio-Rad, USA) to confirm equal loading and transfer, before being blocked in PBST (0.1% Tween-20 PBS) containing 5% (w/v) non-fat dry milk. Subsequently, the membranes were probed with primary antibodies (Supplementary Table 1, online resource) overnight at 4 °C. The membranes were washed for 10 min three times in PBST and then incubated with horseradish peroxidase-conjugated secondary antibodies (Supplementary Table 1, online resource) for 2 h at room temperature. Membranes were washed as previously stated and enhanced chemiluminescence was detected using ECL (MilliporeSigma, USA). Standardised short exposure times were chosen for the analysis of our WB data to minimize any chemiluminescent saturation effect. The protein level was semi-quantified with the measurement of signal intensities using Image lab software (Image Lab Version 6.1ILSPC-V-6-1, Bio-Rad, USA). Total protein was used for normalization (Supplementary Fig. 2, online resource) to avoid tissue and age variability in housekeeping proteins [36, 43].

### Statistical analyses

Quantifications were completed by investigators blinded to the image origins. All statistical analyses were performed using IBM SPSS (SPSS Inc., Chicago, IL, USA, version 26) and R Statistical Software (version 4.0.0, R Core Team, 2013) on RStudio (version 1.2.1335, RStudio Team, 2018). Statistical significance was set at *p* < 0.05 with protected testing for multiple comparisons used.

To assess motor cortex and tract abnormalities in silent and prodromal motor PD, we compared the FA images in two ways: (a) FA projected onto the WM skeleton (skeletonized FA); and (b) original FA whole-brain images. We used the Threshold-Free Cluster Enhancement (TFCE) embedded in the FMRIB Software Library (v6.0) for multiple comparison correction with 10,000 permutations. Skeletonized FAs were compared across visits (baseline versus follow-up) using voxel-wise independent-samples *t* tests, while skeletonized and non-skeletonized FAs were compared between groups at follow-up (iRBD versus controls) using voxel-wise independent-samples *t* tests.

To determine significant motor cortex and tract pathologies in motor PD, differences in coherency, intensity, and diameter of axons between controls and PD groups were analysed using a Kruskal–Wallis test followed by Dunn’s multiple comparison post-hoc test. Group differences (controls versus PD group/s) in other quantitative variables (cell numbers/size, corrected intensity of proteins) were assessed using multivariate analyses covarying for age, post-mortem delay, and gender. Post-hoc Mann–Whitney or Dunn’s multiple comparison tests were used. Spearman correlations were performed to identify significant associations between examined protein levels, αSyn pathology, OLG components and demographic variables.

## Results

### Abnormalities in the motor cortex and tracts in silent and prodromal motor PD

**Fig. 1:**
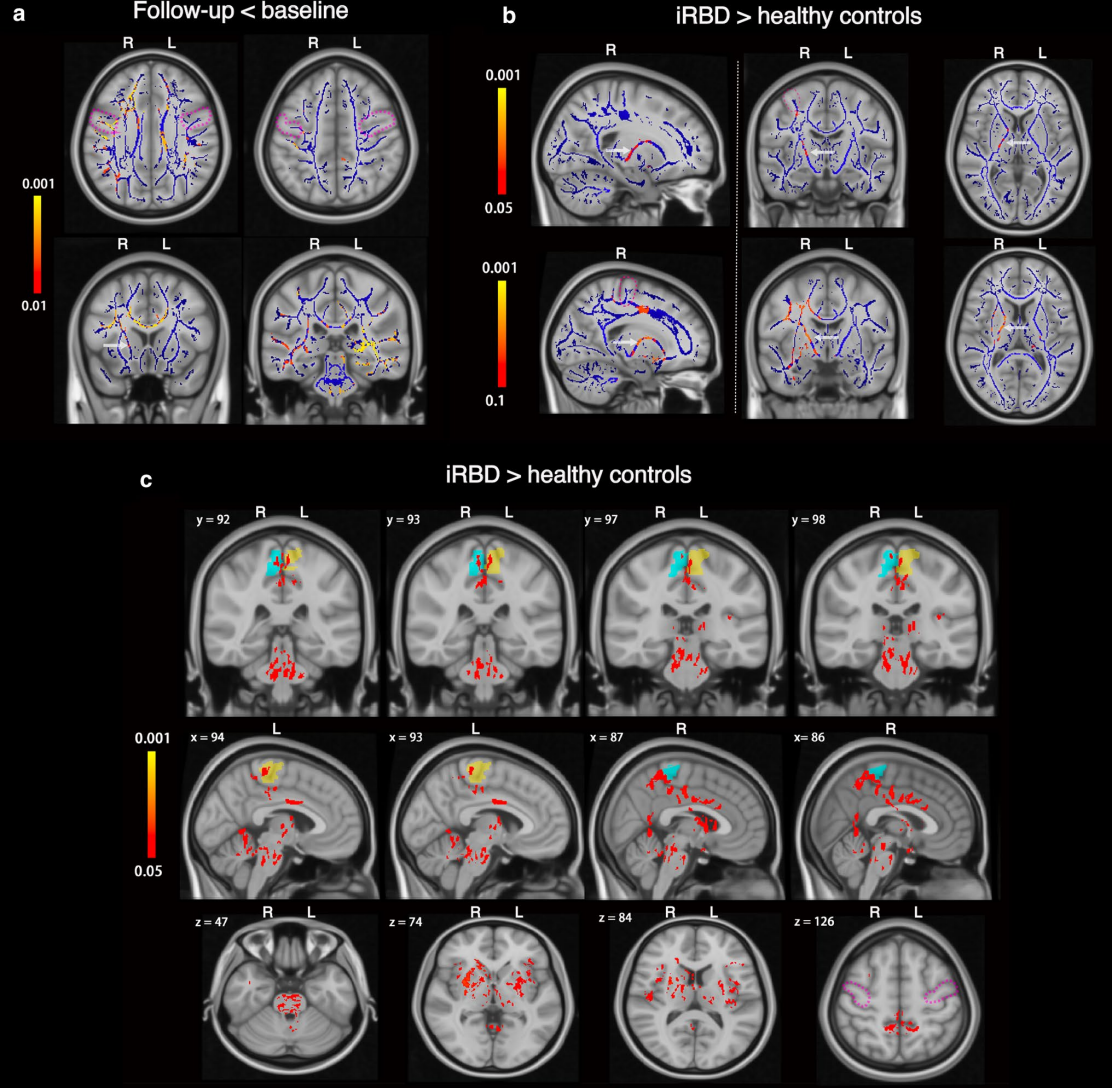
Microstructural changes in the motor system of longitudinally followed iRBD cases that progressed to silent and/or prodromal motor PD. **a** Longitudinal fractional anisotropy (FA) changes in the iRBD group (*n* = 8). Blue lines illustrate the mean skeletonized FA of WM across the two visits. White arrows point to the anatomical location of the cortico-spinal tract. Colour bars represent corrected *p* values (Follow-up < baseline) by threshold-free cluster enhancement with 10,000 permutations. No significant voxel was found for Follow-up > baseline. **b** Skeletonized FA comparison between iRBD and controls at follow-up. Blue lines illustrate the mean skeletonized FA of WM across the two groups. Colour bars represent corrected *p* values for iRBD > healthy controls by Threshold-Free Cluster Enhancement with 10,000 permutations. The threshold *p* value for comparison of the upper row was 0.05 and of the lower row was 0.1. No significant voxel was found for iRBD < healthy controls. **c** Whole-brain FA comparison between iRBD and controls at follow-up. Blue and yellow shadows indicate right and left paracentral lobules, respectively, which contain the primary motor cortex sampled in our post-mortem study. Colour bar represents the corrected *p* values for iRBD > healthy controls by Threshold-Free Cluster Enhancement with 10,000 permutations. No significant voxel was found for iRBD < healthy controls. x, y, and z are MNI (Montreal Neurological Institute) coordinates in the standard space. x ranges from 0 to 181 as from right to left. y ranges from 0 to 217 as from posterior to anterior. z ranges from 0 to 181 as from bottom to top. White arrows in **a** and **b** point to the anatomical location of the cortico-spinal tract. Abbreviations: Follow-up < baseline: iRBD group had decreased FA in the longitudinal analysis, Follow-up > baseline: iRBD group had increased FA in the longitudinal analysis, iRBD > healthy control group: iRBD group had higher FA than that of healthy control group, iRBD < healthy control group: iRBD group had lower FA than that of healthy control group

Comparison of the skeletonized FA longitudinally in the iRBD cases revealed reduced FA in the subcortical motor WM of the precentral gyrus as well as in the superior longitudinal fasciculus and corpus callosum (red-yellow in Fig. 1a, corrected *p* < 0.01), suggesting deteriorating axonal integrity and/or myelination in motor and other WM tracts during the conversion period to motor PD. Follow-up comparison between iRBD and controls showed bilateral loss of DAT binding (as expected, see representative PET images and data in Supplementary Fig. 3, online resource) as well as significantly higher skeletonized FA in the right corticospinal tract during the conversion period to motor PD (Fig. 1b). While all subjects were right-handed, there were no differences between left and right basal ganglia DAT binding dysfunction (between sides *p* = 0.71 for caudate and *p* = 0.90 for putamen). The selective change in the right corticospinal tract may imply that structural modification firstly occurs to compensate for the control of the less motor skilled and used left-sided limbs. In the whole brain FA comparisons, the iRBD cases had higher FA values than controls, concentrating bilaterally in the paracentral lobules, but also seen in the middle cingulate cortex, basal ganglia, cerebellum, and pons (Fig. 1c). Overall, there is evidence of early DTI abnormalities in the motor cortex and WM tracts during the conversion period to motor PD and before a diagnosis of PD can be made.

### Neurofilament changes in white matter axons underlying the motor cortex in motor PD

**Fig. 2:**
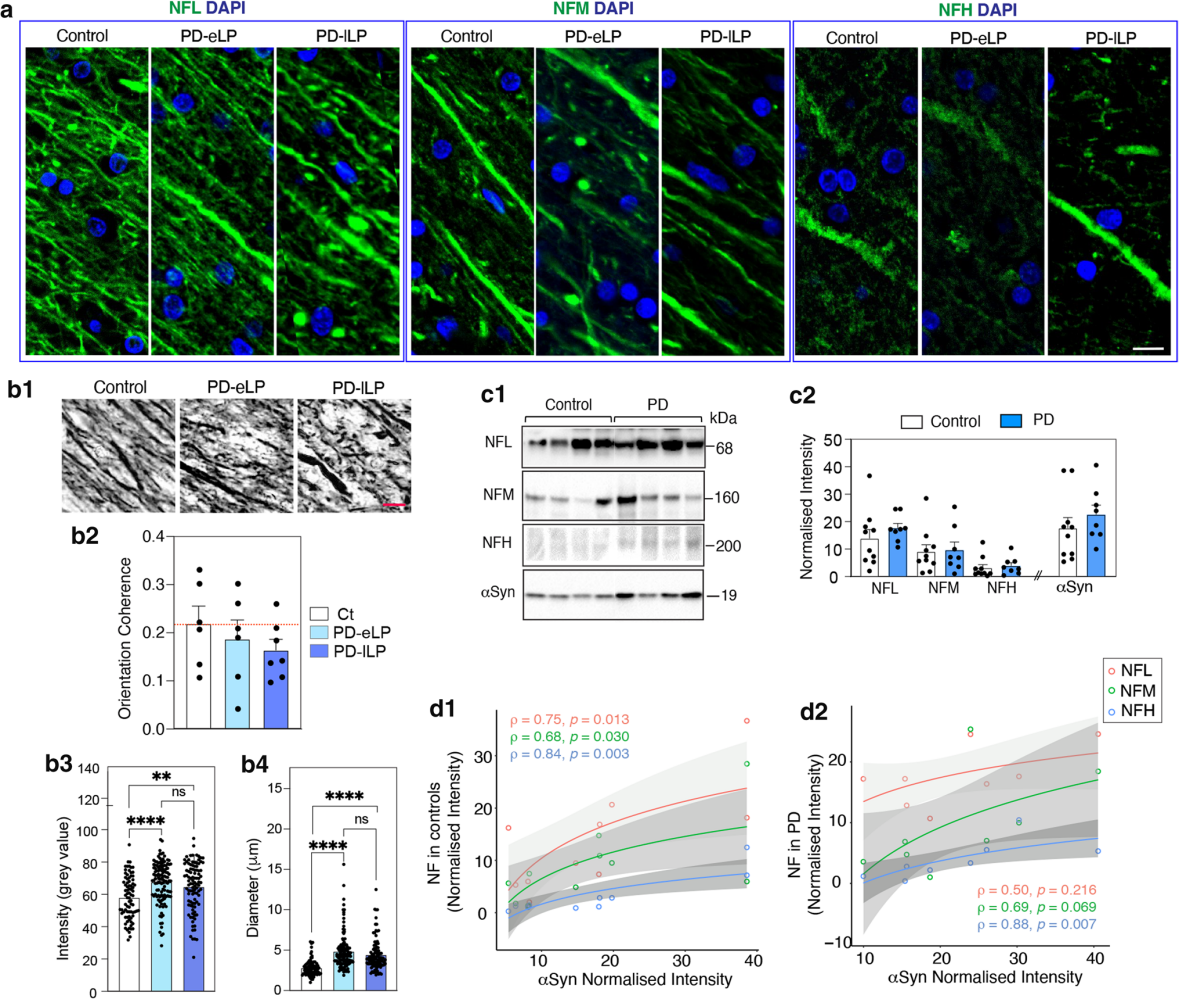
Neurofilament abnormalities in the white matter underlying the motor cortex in motor PD. **a** Immunofluorescence images of axonal morphology (green) identified with neurofilament light (NFL), medium (NFM) and heavy chain (NFH) with glial nuclei labelled with DAPI (blue) in a representative control and early and late PD case (PD-eLP and PD-lLP, respectively). Scale bars are 10 mm for **a** and **b1**. **b1** Representative images of NFL labelled white matter axons used for analysing (mean ± SEM), **b2** axon orientation coherence (immunoperoxidase, n = 6,6,7 for controls, PD-eLP and PD-lLP; dotted red line is the control median), **b3** NFL immunofluorescence intensity (*n* = 4,6,5 for controls, PD-eLP and PD-lLP; 20 samples/case) and **b4** axonal diameter (immunofluorescence, *n* = 4,6,5 for controls, PD-eLP and PD-lLP; 20 samples/case). Kruskal–Wallis test followed by Dunn’s multiple comparisons **p < 0.01, ****p < 0.0001. **c1** Representative western immunoblots and **c2** scatter plots (mean ± SEM) of the relative levels of NFL, NFM, NFH and aSyn in the white matter of controls (*n* = 10) and PD cases (*n* = 8). **d** Positive Spearman rank correlations between the levels of neurofilaments and aSyn in controls (**d1**, *n* = 10), and a loss of the relationship between NFL and aSyn in PD cases (**d2**, *n* = 8). Grey zones indicate 95% confidence intervals which are automatically calculated based on predicted values for the line of best fit

Neurofilaments were used to identify WM axons, with NFL in most axons and relatively rare axons identified using NFH (Fig. 2a). WB confirmed the relative WM levels of the different neurofilaments (NFL > NFM > NFH) and showed no change in these levels between motor PD and controls (Fig. 2c1, c2), although on average the WM axons had increased NFL immunofluorescence in both early and late PD (Fig. 2b3, no difference between PD groups *p* = 0.55). This may be related to the small but significant numbers of axons that had increased diameters in these groups (Fig. 2b1, b4, no difference between PD groups *p* = 0.37). In addition, the alignment of WM axons was more scattered and less aligned in motor PD compared to NFL labelling in controls (Fig. 2b1). Quantitation of the directional coherence showed that using the control median as a reference, 67% of early and 86% of late motor PD cases showed lower axonal coherence (Fig. 2b2). This indicates that, although WM axonal disorganisation in the motor tracts was not a uniform feature in motor PD, it appears to be a progressive change with increasing αSyn stage (Fig. 2b). There was a positive Spearman rank correlation between the levels of WM neurofilaments and less soluble WM αSyn in controls (Fig. 2d1), with a loss of this relationship for NFL and NFM in PD cases (Fig. 2d2). In particular, more NFL was seen in PD cases with low axonal αSyn levels (Fig. 2d2), potentially indicating an earlier change in NFL dynamics prior to increasing αSyn levels. Other cytoskeletal proteins were not increased in motor PD (Supplementary Fig. 4, online resource). Overall, there is evidence for progressive disorientation of WM axons underlying the motor cortex in motor PD prior to increases in αSyn levels.

### Changes in white matter oligodendrocytes and precursors underlying the motor cortex in motor PD

**Fig. 3:**
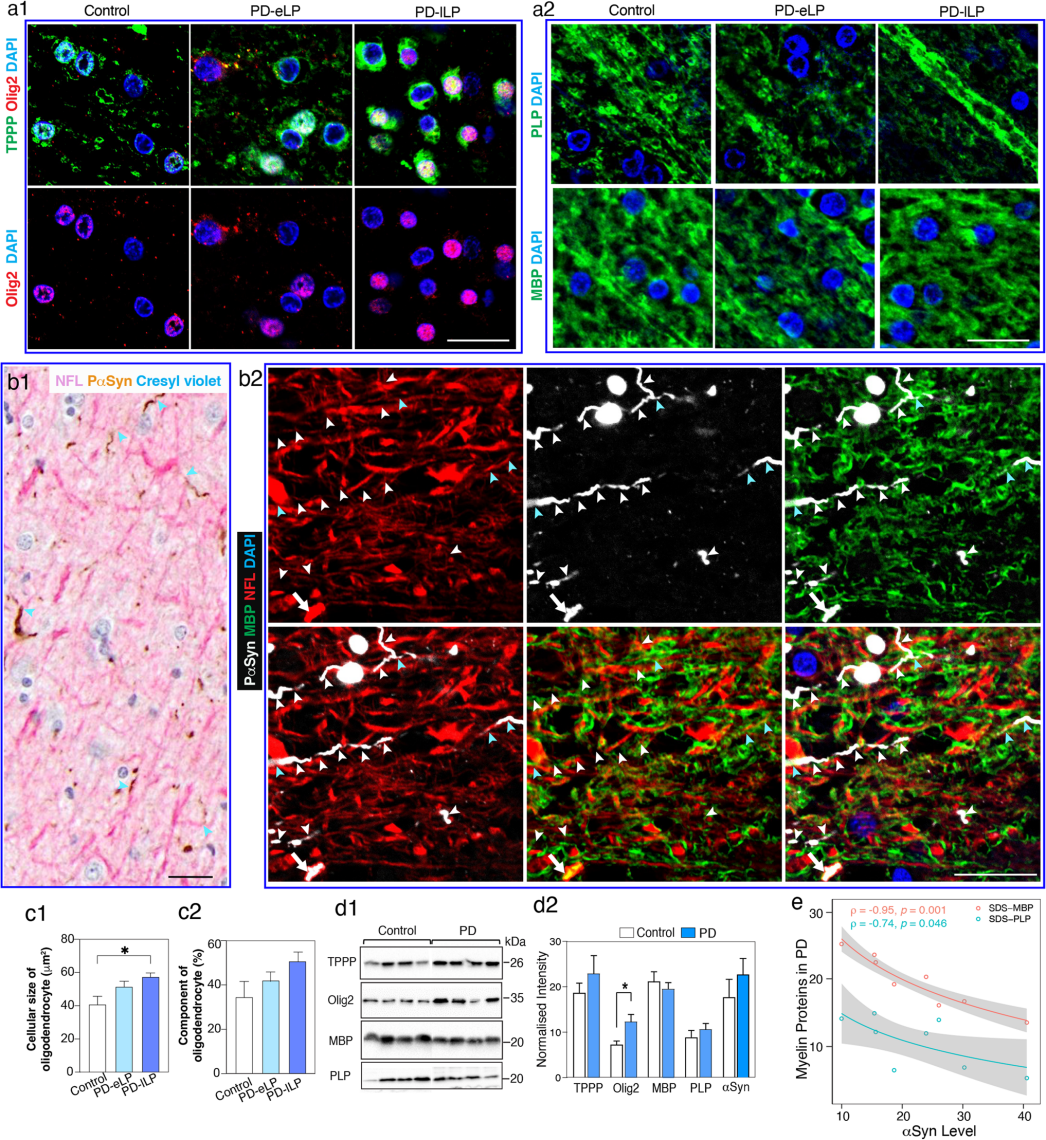
White matter oligodendrocyte, myelin and axonal pathologies underlying the motor cortex in motor PD. **a** Confocal immunofluorescent images from a representative control and early and late PD case (PD-eLP and PD-lLP, respectively) showing the morphology of oligodendrocytes using tubulin polymerization promoting protein (TPPP/P25α, green) and oligodendrocyte transcription factor 2 (Olig2, red) in the left panel (**a1**) and myelin using myelin proteolipid protein (PLP, green) and myelin basic protein (MBP, green) in the right panel (**a2**). Glial nuclei were revealed using DAPI (blue). Scale bars are 20 mm for all images in **a** and **b**. **b1** Neurofilament L (NFL, pink)-labelled axons continuous with phospho-Ser129 aSyn (PS129, brown) labelled neurites were revealed with double labelling IHC (light blue arrowheads). **b2** Immunofluorescence labelling showing the locations of axon (NFL, red), neurites (P-aSyn, white), and myelin (MBP, green). Still forming (blue arrowheads, NFL + , PaSyn + , MBP +) and mature neurites (white arrowheads, PaSyn + but NFL, MBP) as well as a larger congested segment of an axon (white arrows, NFL + , PaSyn + , MBP +) can be seen. **c1** The size and **c2** proportion of TPPP/P25α + oligodendrocytes in each group are shown as bar graphs (mean ± SEM, *n* = 8–13 per group). Statistical difference was tested with the Kruskal–Wallis followed by Dunn’s multiple comparisons. *p < 0.05. **d1** Representative western immunoblots of oligodendrocyte proteins in controls (*n* = 12) and PD cases (*n* = 8). **d2** Column graphs represent the mean of normalised intensity ± SEM. *Mann–Whitney test *p* < 0.05. **e** Negative Spearman rank correlations between the levels of myelin proteins (MBP and PLP) and aSyn in PD cases. Grey zones indicate 95% confidence intervals which are automatically calculated based using the predicted values for the line of best fit

Mature interfascicular OLGs were robustly labelled with TPPP/P25α (Fig. 3a1). Myelin proteolipid protein (PLP) and myelin basic protein (MBP) were expressed as their most abundant myelin proteins (Fig. 3a2 and Supplementary Fig. 5a). OLG precursors were generally smaller and expressed oligodendrocyte transcription factor 2 (Olig2)(Fig. 3a1) [11]. In motor PD, phospho-αSyn segmentally displaced the longitudinal NFL labelling in WM axons underlying the motor cortex (Fig. 3b1, b2), and these axonal regions were not myelinated (Fig. 3b2 and Supplementary Fig. 5b, online resource). These changes were associated with an increase in the average size of WM OLGs in early (26% larger on average) and late (41% larger on average) motor PD compared to controls (Fig. 3c1). There was also an increase in Olig2 but not myelin protein levels in PD (Fig. 3c2, d1, d2), indicating an increase in OLG precursor activity in motor PD. In contrast to axonal NFL, there was a negative correlation between the levels of WM myelin proteins and less soluble WM αSyn in motor PD (Fig. 3e), indicating some demyelination with increasing WM αSyn. This was confirmed by a decrease in the ratio of WM MBP to NFL in motor PD compared to controls (0.76 ± 0.29 versus 1.29 ± 1.19, *p* = 0.08 as shown in Supplementary Fig. 5c, online resource). Overall, there is evidence for a progressive reduction of myelin proteins in association with an increase in OLG precursors and mature OLG size as αSyn accumulates in motor tract axons over the course of motor PD.

### Lewy pathology and neurofilament changes in the motor cortex of motor PD

**Fig. 4:**
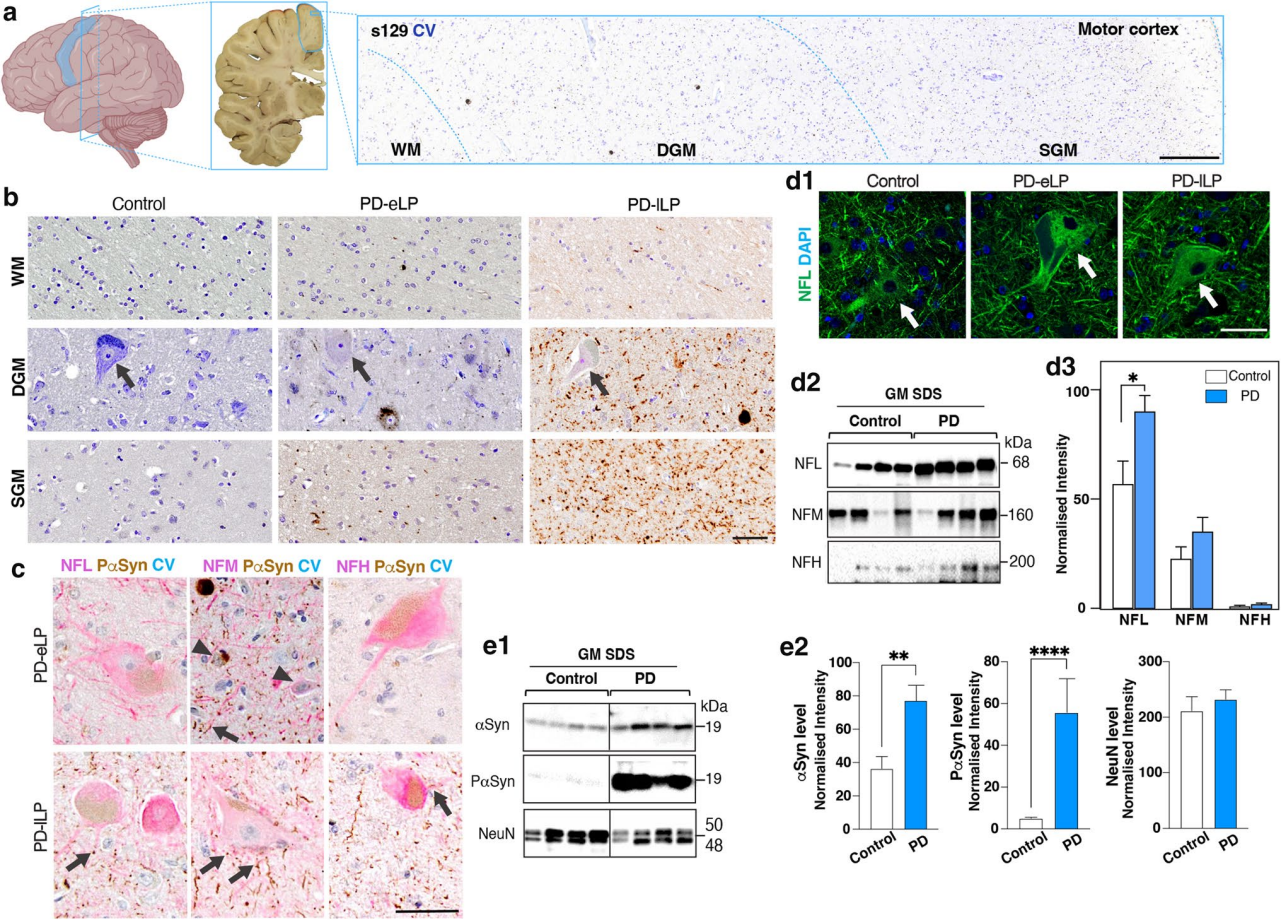
Lewy pathology and neurofilament proteins in the motor cortex in motor PD. **a** Anatomical location of post-mortem primary motor cortex (blue contour) with tissue sampled for pathological studies taken from the medial aspect of the superior surface. The sampled tissue was usually dissected in the coronal slice containing the mammillary body. SGM, DGM, and WM are shown from a formalin-fixed paraffin-embedded section of a representative PD case labelled with s129 (against phospho-Ser129 α-synuclein, brown) and counterstained with cresyl violet (CV, blue); the scale bar represents 200 mm. **b** Regional Lewy pathological load (s129, brown) in SGM, DGM, and WM of representative PD cases compared to a pathologically spared control. Arrows indicate Betz cells; the scale bars represent 50 mm for **b**, **c**, and **d**. **c** Lewy pathology (PaSyn, brown) was mainly found in small neurons (arrowheads, blue CV). In large Betz cells (NFL, pink), Lewy pathology was more dominant in their processes (arrows, PaSyn, brown). **d1** Representative immunofluorescent confocal images of neurofilament light chain (NFL) expressing neurons in control and PD motor cortices. **d2** Representative western immunoblots (SDS-fraction) of neurofilament proteins in the motor cortex of motor PD (*n* = 12) and controls (*n* = 8). d3) Column graphs of the mean ± SEM normalised neurofilament levels in motor cortex. Mann–Whitney test covarying for gender. *p < 0.05. **e1** Representative western immunoblots of pan αSyn (BD-1), phosphorylated αSyn (s129; PαSyn), and NeuN detected in motor cortex (*n* = 8, 12 for controls and PDs, respectively). e2) Column graphs were presented as mean ± SEM. Mann–Whitney tests; **p < 0.01; ****p < 0.0001. *DGM* deep cortical grey matter, *GM* grey matter, *PD-eLP* PD cases with early Lewy pathology (Braak stage 4), *PD-lLP* PD cases with late Lewy pathology (Braak stage 6), *SGM* superficial cortical grey matter, *WM* subcortical white matter. Scale bars are 50 mm for all images unless indicated

Sections of primary motor cortex were sampled from the most medial gyrus in the coronal plane containing the mammillary body (Fig. 4a). Phosphorylated αSyn deposits were observed in motor PD and were absent from controls (Fig. 4b, c), as previously described [24]. Neurites were dominant in SGM and WM, whereas both neurites and neuronal cytoplasmic aggregations (mostly diffuse, beads-like, solid or less often halo-like) were evident in DGM (cortical layers 5–6) (Fig. 4b). In deep cortical layer 5, Betz cells were often observed, but these cells rarely had copious αSyn aggregations in their cytoplasm, unlike those neighbouring smaller pyramidal neurons (Fig. 4c). Neurofilament labelled pyramidal neurons ranged in size from small to large (~ 25–110 mm) with neurofilaments not obviously incorporated into Lewy or other pathologies (Fig. 4c, d1). However, there was a large increase in the levels of NFL in the motor cortex (Fig. 4d2, d3), indicating either an upregulation of the protein and/or a slowing of its metabolism potentially to buttress the axonal changes occurring in the WM. As expected, there was an increase in less soluble grey matter (GM) αSyn while neuronal NeuN remained constant (Fig. 4e1, e2), indicating no neuronal loss in the motor cortex of motor PD but increased protein levels per neuron. The neuronal phosphorylation of αSyn was upregulated in PD compared to controls (Supplementary Fig. 6, online resource). The increases in NFL and αSyn levels in motor cortex in motor PD were not correlated, indicating a similar disconnect to that observed in the WM underlying the motor cortex in the same cases. Overall, motor cortex neurons have an increase in local NFL and αSyn in response to motor PD.

### Changes in oligodendrocytes and precursors in the motor cortex of motor PD

**Fig. 5:**
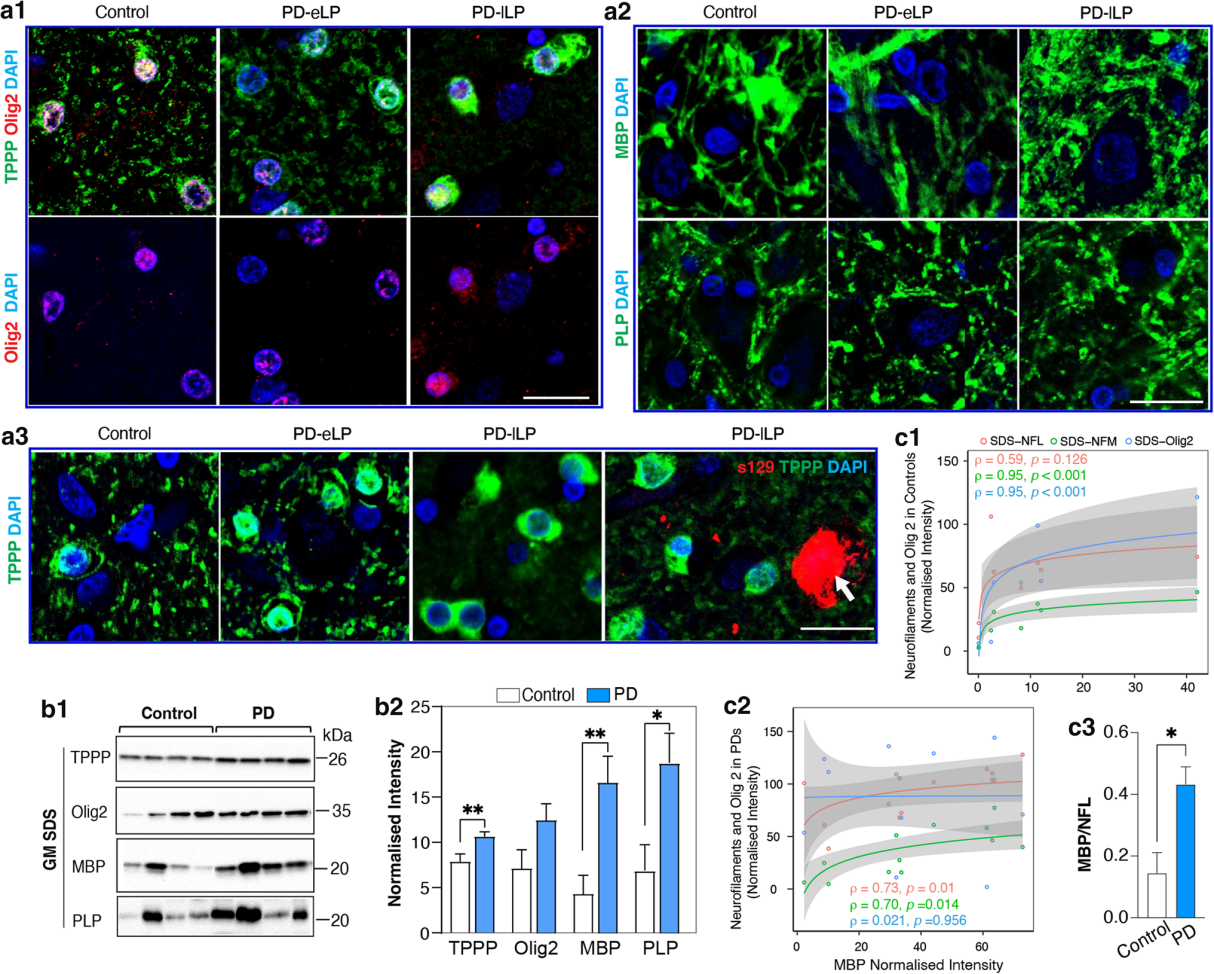
Grey matter oligodendrocyte and myelin protein abnormalities in the motor cortex in motor PD. **a** Representative confocal images showing oligodendrocyte morphology using tubulin polymerization promoting protein (TPPP/P25α, green) and oligodendrocyte transcription factor 2 (Olig2, red) in the left panel (**a1**) and myelin using myelin proteolipid protein (PLP, green) and myelin basic protein (MBP, green) in the right panel (**a2**). **a3** TPPP and s129 (against phospho-Ser129 α-synuclein) colabelling revealed inclusions in neurons (white arrow) but not in oligodendrocytes in the deep grey matter of PD. PD-eLP: PD with early Lewy pathology, PD-lLP: PD with late Lewy pathology. Scale bars are 20 mm for all images. **b1** Representative western immunoblots of the oligodendrocyte proteins in the SDS fraction of motor cortex in PD (*n* = 12) and controls (*n* = 8). **b2** Column graphs represent the mean ± SEM normalised levels for these proteins in PD (*n* = 12) and controls (*n* = 8). Mann–Whitney test covaried for gender. *p < 0.05, **p < 0.01. **c1** Positive Spearman rank correlations between the levels of MBP and NFM or Olig 2 and not NFL in controls (*n* = 8) and **c2** between MBP and these neurofilaments and not Olig 2 in the PD group (*n* = 12). Grey zones indicate 95% confidence intervals automatically calculated based on the predicted values for the line of best fit. c3) The ratio of the myelin protein MBP to the axonal protein neurofilament light chain (NFL) in PD (*n* = 12) and controls (*n* = 8). Mann–Whitney test covaried for gender. *p < 0.05

OLGs in cortex have diverse functions (including perineuronal satellite OLGs [61]) with many axons unmyelinated (only initial axon segments of inhibitory neurons are routinely myelinated in GM [58]) and the morphological changes observed in WM OLGs in motor PD were less obvious in cortical OLGs in the same cases (Fig. 5a1). There was no increase in OLGs size (Supplementary Fig. 7a, online resource), no change in MBP and PLP labelled myelin structures (Fig. 5a2) and no intracellular αSyn deposits observed in cortical OLGs or in association with neuronal αSyn accumulation (Fig. 5a3). The proportion of oligodendrocytes was increased in the DGM but not in the SGM in motor PD (Supplementary Fig. 7b) with no increase in Olig2 + precursors (Fig. 5a1, b1, b2). In contrast to WM OLGs, there was a significant increase in the cortical levels of TPPP/P25α, MBP and PLP in motor PD compared to controls (Fig. 5b1, b2), indicating an upregulation of myelinating activity of mature cortical OLGs in motor PD. The increase in MBP levels correlated with increased NFL levels in PD and not controls (Fig. 5c1, c2) with the comparative levels of MBP to NFL nearly threefold greater in motor PD (Fig. 5c3). This indicates a greater increase in cortical myelination associated with the local increase in neuronal NFL in motor PD. In controls, there was a positive correlation between MBP and Olig2 levels that was lost in motor PD (Fig. 5c1, c2), with more Olig2 in motor PD cases with low MBP levels (Fig. 5c2). Overall, motor cortex OLGs significantly upregulate their myelinating ability compared to WM OLGs in response to the significant increase in cortical NFL in motor PD.

## Discussion

Compared with the continued degeneration of the nigrostriatal system through the silent, prodromal and motor stages of PD, until recently the motor cortex and tracts have been thought to be largely spared in PD [8]. Our study confirms no loss of neurons in the motor cortex up to the late stages of PD despite changes in excitability (overall hypoactive [26]). In the absence of outright neurodegeneration of motor cortex neurons by end-stage PD, the changes observed should be considered adaptive structural changes to motor PD. Our data show that such adaptive structural changes occur in the neurons and OLGs of the motor cortex as well as in their axons and supporting OLGs in the underlying WM. At the neuronal level, there is evidence of αSyn accumulation in WM axon segments that are devoid of both NFL and myelin sheath covering, more disorientation of intact motor tract axons, and an increase in axonal and cytoplasmic NFL levels without obvious cytoskeletal changes in motor cortex pyramidal neurons in motor PD. αSyn deposition occurs in the motor cortex mainly in the form of Lewy neurites which may be axonal and/or dendritic and increase with disease progression. A surprising and highly novel finding was the diverse impact of these structural changes on the different types of OLGs in response to motor PD–a mild demyelinating phenotype in motor tract WM with evidence of OLG replenishment by activated precursors that correlate with αSyn accumulation in axon segments, and a hypermyelinating phenotype in the overlying motor cortex in response to increased cortical NFL. The identification of these novel structural adaptions in motor circuits in motor PD is likely due to activity changes in motor networks, as our DTI study shows increased FA in motor cortices and WM tracts in concert with the initiation of degenerative changes in the nigrostriatal tract and prior to substantive motor symptoms. These data confirm major adaptive structural changes in brain motor circuits are an integral part of motor PD.

### Adaptive axonal changes and αSyn deposition in motor tracts in motor PD

The αSyn accumulation in axons in WM motor tracts was unexpected, although extensive axonal Lewy neurites are a prevalent feature of PD associated with regions of high Lewy pathology load [10] (e.g. see Supplementary Fig. 5b, online resource). The density of axonal αSyn accumulations in WM motor tracts was lower and thinner than the motor cortex neurites observed, increasing in frequency with disease progression (Fig. 4b). Previous evidence suggests a retrograde accumulation of αSyn in axons [45, 65], although in animal experiments the evidence suggests accumulation in axons is due to perturbations in anterograde axonal transport of αSyn [1, 62]. This is more consistent with the axonal αSyn accumulations that are most frequently observed at axonal branches surrounded by neurofilaments and the concept that these accumulations are initiated at axon collaterals [28]. The concept that αSyn plays a role in axon growth has been suggested based on cultured cortical neurons from A53T transgenic mice that have elongated axons with enhanced arborizations and WM tracts with higher densities of thinner axons [55, 64]. If αSyn is involved in axon arborization, then the loss of myelination we observed at these sites would assist with the collateralization by relieving the myelin restriction as observed experimentally [46]. αSyn has been shown to inhibit OLG maturation and the formation of myelin sheaths [22], consistent with our findings of increased OLG precursors, myelin protein reduction with increasing WM αSyn levels, and myelin sheath loss around axonal αSyn accumulations. Hyperbranching of axons is a well-known anatomical commonality of Lewy-prone neurons in PD [65], neurons with naturally enhanced levels of αSyn that include lower layer cortical pyramidal neurons [41]. This may suggest a more important role for αSyn in the plasticity of axonal branching, and thus the accumulation of αSyn in the WM motor axons from intact neurons in the motor cortex may indicate an enhanced collateralization process to ensure maximum delivery of any stimulus from a more hypoactive motor cortex. Such an adaptive structural change does not appear to be degenerative to WM motor tracts.

The intact WM motor tract axons identified by NFL-immunopositivity had less coherence and parallel orientation in the majority of cases with motor PD, potentially consistent with an increase in axonal collateralisation within the tract. The WM NFL levels were enhanced in motor PD patients, particularly with low axonal αSyn levels, suggesting that these changes in axonal NFL occur prior to the axonal αSyn accumulation. Neurofilaments are essential for the radial growth and structural stability of myelinated axons with NFL as their main structural neurofilament [21, 69]. Ablation of NFL reduces axon calibre and excitatory synaptic currents in cultured human motor neurons [52] and delays the maturation of regenerating myelinated axons in transgenic mice [72], while overexpression increases the density of NFL neurofilaments in myelinated axons [40]. At the onset of typical motor PD, there is an increase in fibre bundle size in the corticospinal tract [4] and a continuing increase with disease progression [37]. Our study shows increased axonal NFL intensity levels in association with an increase in axon diameter in motor PD, and a rearrangement of NFL-immunopositive axons in association with segmental αSyn accumulation in WM motor tracts over time. This is consistent with rearrangements and growth of axonal sprouts, and this change may increase the WM motor tracts over time in response to changes in network activity.

### Adaptive structural cortical changes and αSyn deposition in motor PD

There was no overall loss of neurons in the motor cortex in PD, consistent with previous studies [24], but there was a significant increase in NFL and αSyn levels. The degree of αSyn accumulation over time in the motor cortex was somewhat surprising, but consistent with increased sensitivity of newly available αSyn antibodies. Some pathology was observed at an early PD stage but significant neuritic pathology was mainly seen with disease progression. Neuronal cytoplasmic Lewy inclusions were predominantly found in cortical layers 5&6 which contain intratelencephalic (layer 5a), corticospinal and corticostriatal (layer 5b), and fusiform corticothalamic (layer 6) pyramidal neurons [38], suggesting αSyn accumulation concentrates in cortical pyramidal neurons projecting axons into the WM motor tracts. The motor cortex neurons are some of the largest pyramidal neurons in the brain and neurofilaments are abundant in their large dendritic trees [33, 70]. Increased neuronal NFL increases dendritic arborization and reductions in neuronal NFL decrease dendritic arborization in transgenic mice, with lower ratios of NFH and NFM to NFL critical for the growth of complex dendritic trees in motor neurons [33]. Activity levels are the main driver shaping dendritic arbors with in vivo animal studies revealing that more static dendritic trees occur in more electrically active motor neurons and an increase in dendritic filopodia occurs with genetic reduction of activity [31]. These studies suggest that the NFL increase in motor cortex in motor PD is likely in response to changes in synaptic input to these lower layer pyramidal neurons requiring an increase in their dendritic arborisation. Again, such changes appear to be adaptive structural changes rather than associated with a degenerative loss of motor cortex neurons.

High NFL levels in peripheral biofluids (serum, plasma or CSF) are considered a biomarker for axonal degeneration, and there is considerable evidence that motor dysfunction in PD relates to high peripheral NFL levels that occur in concert with nigrostriatal degeneration [6, 25]. However, nigrostriatal degeneration occurs largely prior to motor symptom onset [32] and the compensatory NFL changes observed in motor cortex could contribute to any increase in NFL levels with disease progression in a similar way to the increases in peripheral NFL levels identified in longer-term recovering stroke patients [47, 60]. In these patients the increase in peripheral NFL levels has been shown to be predictive of improving rather than declining function [47, 60]. Higher plasma NFL levels in motor PD are independent predictors of depression and anxiety [68], suggestive of a potential correlate that is not strictly related to neurodegeneration. It will be important in future studies to relate brain tissue NFL levels to those found in peripheral biofluids to determine and monitor these structural disease-related changes.

### Oligodendrocyte responses in motor PD

It is probably not surprising that the neuronal structural changes identified impacted on OLGs, but the different responses of WM versus cortical OLGs were a surprise and have not been previously evaluated in motor PD. As indicated above, αSyn inhibits myelin sheath formation [22] with myelin sheaths obstructing axonal branching [46] and αSyn accumulation occurring predominantly at axonal branch points [28]. Our data suggest that the adaptive structural changes in mature WM myelinating OLGs to motor PD are a loss of some myelin sheath segments around new branch site in axons accumulating αSyn and an enlargement of the OLG cell bodies to absorb and reshape their processes. This is consistent with concepts shown in animal studies, but such structural changes have not been identified in human tissues to date [46]. An additional appropriate response to an increase in axonal collateralisation would be an increase in the density of WM OLG precursors, as we observed, as more OLGs would be required to myelinate new collateral axons. Due to the high abundance of OLGs in WM tracts, this increase in size and density over time is likely to increase the overall WM fibre bundle size, as observed in DTI studies [4, 37].

In contrast to myelinating WM OLGs, OLGs in the motor cortex made more myelin (increased protein levels) in response to the increase in cortical NFL. Cortical perineuronal satellite OLGs are mainly distributed in lower cortical layers where they attach closely to and metabolically support pyramidal neurons [61]. An increase in the surface area of more hypoactive pyramidal neurons (see above) would require an increase in OLG processes. In addition, intracortical fast-spiking inhibitory interneurons are the most abundant inhibitory interneurons concentrating in layers 4, 5 and 6, and are frequently myelinated [58, 63]. They are responsible for short intracortical inhibition of pyramidal neurons by synapsing on pyramidal axonal initial segments as well as their dendrites [63] and increase their axonal aborisation and myelination in response to increased activity [58]. Reduced effectiveness of short intracortical inhibition in the motor cortex is the most reliable abnormality identified in motor PD thought to be due to saturated activity of these neurons [17, 23], with dopamine input modulating this inhibitory network [13]. An increase in cortical myelination could indicate increased arborization and activity of such motor cortex interneurons which would result in increased inhibition to and hypoactivity of their targeted pyramidal neurons, and also parallel an increase in NFL and the dendritic arbor of their targeted neurons. As speculated above, if αSyn is involved in axonal collateralisation, then the increase in Lewy neurites in the motor cortex may represent the increased axonal branching of these inhibitory interneurons. Further assessment of animals that model these changes would be of value to understand better how these adaptive structural changes occur and their potential to impact functionally on motor PD.

### Timing of the adaptive structural changes in motor cortex and tracts in PD

Corticospinal tract involvement in early diagnosed motor PD using DTI is well established with the increased FA considered to be compensatory rather than degenerative [4, 5, 39], consistent with our findings. In terms of microstructure, there are a number of structural changes we observed in WM motor tracts histologically that are likely to contribute to increased FA and overall fibre bundle size, including αSyn accumulation, axon collateralisation, and more OLGs of larger size. To identify when these adaptive structural changes in the motor WM tracts begin, we assessed motor tracts in iRBD patients progressing to the preclinical silent and prodromal motor PD stages using DTI and identified that the increased DTI occurred in concert with nigrostriatal loss prior to the diagnosis of PD. Longitudinally, these iRBD patients had a reduction in FA from baseline to follow-up when they had increased FA compared to controls, suggesting that at baseline prior to nigrostriatal degeneration there was already an increase in FA. Although speculative it appears that changes in motor circuit excitability leads to extremely early microstructural tract changes in vulnerable iRBD patients that contributes to the later onset of motor PD. iRBD is considered a synucleinopathy but it is unclear when and where αSyn begins to deposit in those iRBD patients that convert to a neurodegenerative condition. The present data could suggest that adaptive structural changes in neurons with highly branching axonal arbors that change their excitability in iRBD are most likely to accumulate αSyn. This would include motor cortical neurons that are abnormally active during REM sleep.

Our study has a number of strengths and weaknesses. A strength is the number of techniques used to quantitatively assess changes in the motor cortex and WM tracts allowing for robust confirmation of results using multiple different techniques in 48 patients with PD at different motor stages and 48 matched controls. A weakness is the number of patients studied at each motor stage but a strength is the identification of similar changes across the entirety of the different clinical phases of motor PD, and the inclusion of a longitudinal study of iRBD patients that converted to silent and/or prodromal motor PD. These cohorts revealed the same structural changes that increased with disease progression, providing some replication over the disease course as well as replicating findings from DTI data in early PD cases. While this gives additional confidence to the timing of the adaptive structural changes observed, further replication is required as these changes have not been previously histologically assessed or analysed in human tissue.

## Conclusions

Our study fills a gap in knowledge on the structural changes observed in the motor cortex and its WM tracts in motor PD by identifying when such changes begin (just prior to nigrostriatal degenerative changes) and the cellular elements they represent (increased NFL, αSyn accumulation, axon collateralisation, increased myelinating OLGs). A surprise was the accumulation of αSyn in axonal segments that suggests a role for αSyn in assisting with focal demyelination at axonal branch points for the formation of axon collaterals in these and probably other hyperbranching axons. A further surprise was the different response of OLG in the motor cortex versus WM motor tracts which may reflect the calibre of axons the different OLGs myelinate (small, finely branched intracortical axons of inhibitory interneurons versus very large pyramidal projection neurons). OLGs produce an intrinsic length of myelin sheath [7] with the changes observed suggesting that the cortical OLGs increase their myelin to wrap more small inhibitory axon collaterals in their vicinity, while WM OLGs increase their number and size to wrap more large pyramidal axon collaterals in the WM motor tracts. These structural changes occur without neuronal loss and appear to be an adaption to changes in motor cortex excitability. Importantly, over the course of PD these adaptive structural changes affect more and more cortical and WM cells, making it increasingly likely that they may also impact on motor symptoms in PD.

## Supplementary Information

Below is the link to the electronic supplementary material.

Supplementary file1 (PDF 3087 KB)

## Abbreviations

18F-FP-CIT PET: 18F-N-(3-fluoropropyl)-2β-carbon ethoxy-3β-(4-iodophenyl) nortropane positron emission tomography
AP: Alkaline phosphatase
αSyn: Alpha-synuclein
BCA: Bicinchoninic acid
BSA: Bovine serum albumin
CSF: Cerebrospinal fluid
DAPI: 4′,6-Diamidino-2-phenylindole
DAT: Dopamine transporter
DGM: Deep cortical grey matter
dMRI: Diffusion magnetic resonance imaging
DTI: Diffusion tensor imaging
EEG: Electroencephalography
FA: Fractional anisotropy
FFPE: Formalin-fixed paraffin-embedded
FOV: Field of view
FWHM: Full-width half-maximum
GDC: Gradient distortion correction
GM: Grey matter
HIAR: Heat-induced antigen retrieval
HRP: Horseradish peroxidase
IHC: Immunohistochemistry
IF: Immunofluorescence
iRBD: Idiopathic rapid-eye-movement sleep behaviour disorder
MBP: Myelin basic protein
MDS-UPDRS: Movement disorder society unified Parkinson’s disease rating scale
MMSE: Mini-mental state examination
MNI: Montreal neurological institute
MoCA: Montreal cognitive assessment
MPRAGE: Magnetization-prepared rapid acquisition gradient-echo
MRI: Magnetic resonance imaging
NFL: Neurofilament light chain
NFM: Neurofilament medium chain
NFH: Neurofilament heavy chain
OLG: Oligodendrocyte
Olig2: Oligodendrocyte transcription factor 2
OSEM: Ordered subset expectation maximization
PBS: Phosphate buffered saline
PD: Parkinson’s disease
PLP: Myelin proteolipid protein
PSG: Polysomnography
REM: Rapid-eye-movement
RBDSQ: IRBD screening questionnaire
ROI: Region of interest
SCOPA-AUT: Scale for outcomes in PD-autonomic
SDS: Sodium dodecyl sulphate
SGM: Superficial cortical grey matter
TBS: Tris buffered saline
TBSS: Tract-based spatial statistics
TFCE: Threshold-free cluster enhancement
TPPP: Tubulin polymerization promoting protein
WB: Western immunoblotting
WM: White matter
