# Correction to: Adaptive structural changes in the motor cortex and white matter in Parkinson’s disease

**DOI:** 10.1007/s00401-022-02523-3

**Published:** 2022-11-27

**Authors:** YuHong Fu, Liche Zhou, Hongyun Li, Jen‑Hsiang T. Hsiao, Binyin Li, Onur Tanglay, Andrew D. Auwyang, Elinor Wang, Jieyao Feng, Woojin S. Kim, Jun Liu, Glenda M. Halliday

**Affiliations:** 1 Brain and Mind Centre & Faculty of Medicine and Health School of Medical Sciences, The University of Sydney, Sydney, NSW 2050 Australia; 2 Department of Neurology and Institute of Neurology, Ruijin Hospital, Shanghai Jiao Tong University School of Medicine, Shanghai, 200025 China; 3 Neuroscience Research Australia & Faculty of Medicine School of Medical Sciences, University of New South Wales, Sydney, NSW 2052 Australia

## Correction to: Acta Neuropathologica (2022) 144:861–879 10.1007/s00401-022-02488-3

In this article author would like to make few changes in the article text. The corrections are given below.

1. In the Section of Materials and methods, under the heading “Immunohistochemistry and immunofluorescence” on page 865, the 12th line of the right column text should be corrected as “1 μg/ml”.
2. In the Section of Results, under the heading “Lewy pathology and neurofilament changes in the motor cortex of motor PD” on page 871, the 15th line of the left column text should be corrected as “(~ 25–110 μm)”.
3. In the Section of Results, Figure 2 legend on page 869, the 6th line of the left column text should be corrected as “10 μm”; the 5th, 8th, and 9th lines of the right column text should be corrected as “αSyn”.
4. In the Section of Results, Figure 3 legend on page 871, the 10th line of the left column text should be corrected as “20 μm”; the 11th and 27th lines of the left column texts should be corrected as “αSyn”; the 14th line of the left column text should be corrected as “PαSyn”; the 16th and 18th lines of the left column texts should be corrected as “PαSyn+”.
5. In the Section of Results, Figure 4 legend on page 872, the 10th line of the left column text should be corrected as “200 μm. Brain icon in a was created with BioRender.com.”; the 13th line of the left column texts should be corrected as “50 μm” and “PαSyn”, respectively; the 16th line of the left column text should be corrected as PαSyn”; the 14th line of the right column text should be corrected as “50 μm”.
6. In the Section of Results, Figure 5 legend on page 873, the 11th line of the left column text should be corrected as “20 μm”.

