# Supplementary material for: Adaptive structural changes in the motor cortex and white matter in Parkinson’s disease
Source: Acta Neuropathol. 2022 Sep 2;144(5):861–79. doi: 10.1007/s00401-022-02488-3 (PMC9547807; doi:10.1007/s00401-022-02488-3)
Supplement: Supplementary file 1 — Supplementary file1 (PDF 3087 KB) [file 401_2022_2488_MOESM1_ESM.pdf]

Online Supplementary Resource for

Adaptive structural changes in the motor cortex and white matter in Parkinson's disease

YuHong Fu[1], Liche Zhou[2], Hongyun Li[1], Jen-Hsiang T. Hsiao[1], Binyin Li[2], Onur Tanglay[3], Andrew D. Auwyang[1], Elinor Wang[1], Jieyao Feng[1], Woojin S. Kim[1,3], Jun Liu[2]*, Glenda M. Halliday[1,3]*

[1]*Brain and Mind Centre & Faculty of Medicine and Health School of Medical Sciences, The University of Sydney, Sydney, NSW 2050, Australia*

[2]*Department of Neurology and Institute of Neurology, Ruijin Hospital, Shanghai Jiao Tong University School of Medicine, Shanghai, 200025, China.*

[3]*Neuroscience Research Australia & Faculty of Medicine School of Medical Sciences, University of New South Wales, Sydney, NSW 2052, Australia*

***Correspondence to**: Professor Glenda Halliday, email: glenda.halliday@sydney.edu.au, Tel: +61 2 9351 0888, Fax: +61 2 9114 4224

And

Professor Jun Liu, E-mail: jly0520@hotmail.com; Tel: +8621-64454473

Supplementary Table 1 - Antibodies used in this study

IHC: immunohistochemistry; IF: immunofluorescence

Antibodies Clone, Code	Immunogen/epitope	Identifier (RRID)	Antibody supplier	Methods	Dilution
**Primary antibodies**<br>• Pathology markers					
Mouse Anti-Alpha-synuclein clone 42, 610787	Rat Synuclein-1 aa. 15-123.	AB_398108	BD Transduction	WB	1:2,000
Rabbit Anti-phospho S129 Alpha-synuclein, EP1536Y, Ab51253	Synthetic peptide within Human Alpha-synuclein aa 100 to the C-terminus.	AB_869973	Abcam	WB/IHC/IF	1:2000 /1:1,000
• Neuronal and cytoskeleton proteins					
Mouse anti-NeuN, clone A60, MAB377	Purified cell nuclei from mouse brain	AB_2298772	MerckMillipore	WB	1:1000
Mouse anti-Neurofilament H (NF-H), clone SMI-32, 801701	homogenized hypothalami from Fischer 344 rat brain	AB_2564642	BioLegend	WB/IHC/IF	1:1,000/ 1:200
Mouse anti-Neurofilament M (NF-M), clone RMO 14.9, #34-4000	Rat NF-M	AB_2533154	ThermoFisher	WB/IHC/IF	1:1000/ 1:200
Mouse anti-Neurofilament Light chain (NF-L), clone DA2, #13-0400	Specifically recognizes a phosphate-independent epitope on NF-L	AB_2532995	ThermoFisher	WB/IHC/IF	1:1000/ 1:200
Mouse anti-Beta-Tubulin III Antibody, Clone TUJ1, #60052	Rat brain microtubules	AB_1118535	StemCell Tech	WB	1:1000
Mouse anti-Beta-actin Antibody (AC-15), Ab6276	Synthetic peptide corresponding to beta Actin aa 1-100 (N terminal)	AB_2223210	Abcam	WB	1:20000
• Oligodendrocyte and myelin markers					
Goat anti- p25α/Tubulin Polymerization Promoting Protein (TPPP) antibody, poly, PA5-19243	Synthetic peptide sequence (DPSKDRAAKRLSLES) corresponding to the internal amino acids of TPPP	AB_10979400	ThermoFisher	WB/IHC/IF	1:2000/ 1:500
Rat anti- p25α [34]	Recombinant human TPPP/p25α	n/a (no applicable)	Gift of Prof. Poul Henning Jensen (Aarhus University, Denmark)	IF	1:500
Goat anti-Olig2, AF2418	E. coli-derived recombinant human Olig2 (Met1-Lys323)	AB_2157554	R&D	IF	1:100
Mouse anti-myelin proteolipid (PLP), clone plpc1, MCA839G	Synthetic peptide GRGTKF corresponding to C terminal region of myelin proteolipid protein	AB_2237198	Bio-Rad	IF/IHC	1:50/1:100
Rabbit anti-myelin proteolipid (PLP), PA3-150	Synthetic peptide corresponding to residues C (109) GKGLSATVTGGQKGRGSRG(128) of PLP	AB_2299792	Thermofisher	WB	1:2000
Mouse anti- MBP antibody, clone 22, MCA686S	Human myelin basic protein /aa84-89	AB_325006	Bio-Rad	WB/IHC/IF	1:2000/ 1:100

Supplementary Table 1 continued - Antibodies used in this study
IHC: immunohistochemistry; IF: immunofluorescence

**Antibodies Clone, Code**	**Immunogen/epitope**	**Identifier (RRID)**	**Antibody supplier**	**Methods**	**Dilution**
**Secondary antibodies**<br>• HRP (horseradish peroxidase) conjugated 2nd antibodies					
Polyclonal Goat Anti-Rabbit immunoglobulins/HRP, P0448	Immunoglobulins, mainly IgG, isolated from rabbit serum	AB_2617138	Dako	WB	1:2000
Polyclonal Rabbit Anti-Mouse immunoglobulins/HRP, P0260	Immunoglobulins, mainly IgG, isolated from mouse serum	AB_2636929	Dako	WB	1:2000
Polyclonal Rabbit Anti-Rat immunoglobulins/HRP, P0162	Immunoglobulins, mainly IgG, isolated from rat serum	n/a	Dako	WB	1:2000
ImmPRESS® HRP Horse Anti-Mouse IgG Polymer Detection Kit, Peroxidase, MP-7402	Mouse Gamma Immunoglobins	AB_2336528	VectorLabs	IHC	Ready to use
ImmPRESS® HRP Horse Anti-Rabbit IgG Polymer Detection Kit, Peroxidase, MP-7401	Rabbit Gamma Immunoglobins	AB_2336529	VectorLabs	IHC	Ready to use
• AP (Alkaline phosphatase) conjugated 2nd antibody					
ImmPRESS®-AP Horse Anti-Mouse IgG Polymer Detection Kit, Alkaline Phosphatase, MP-5402	Mouse Gamma Immunoglobins	AB_2336535	VectorLabs	IHC	Ready to use
ImmPRESS®-AP Horse Anti-Rabbit IgG Polymer Detection Kit, Alkaline Phosphatase, MP-5401	Rabbit Gamma Immunoglobins	AB_2336536	VectorLabs	IHC	Ready to use
• Alexa Fluor labelled 2nd antibodies					
Donkey Anti-Goat IgG-647, A-21447	Goat Gamma Immunoglobins	AB_2535864	ThermoFisher	IF	1:250
Donkey Anti-Mouse IgG-488, A-21202	Mouse Gamma Immunoglobins	AB_141607	ThermoFisher	IF	1:250
Donkey Anti-Rabbit IgG-568, A-10042	Rabbit Gamma Immunoglobins	AB_2534017	ThermoFisher;	IF	1:250
Donkey Anti-Mouse IgG-647, A-31571	Mouse Gamma Immunoglobins	AB_162542	ThermoFisher	IF	1:250
Donkey anti-Rat IgG (H+L) Highly Cross-Adsorbed Secondary Antibody, Alexa Fluor 488, A21208	RAT Gamma Immunoglobins Heavy and Light chains	AB_2535794	ThermoFisher	IF	1:250
Goat anti-Mouse IgG1 Cross-Adsorbed Secondary Antibody, Alexa Fluor 568, A-21124	Mouse IgG gamma 1	AB_2535766	ThermoFisher	IF	1:250
Goat anti-Mouse IgG2a Cross-Adsorbed Secondary Antibody, Alexa Fluor 488, A-21131	Mouse IgG 2a	AB_2535771	ThermoFisher	IF	1:250
Goat anti-Mouse IgG2a Cross-Adsorbed Secondary Antibody, Alexa Fluor 647, A-21242	Mouse IgG 2b	AB_2535811	ThermoFisher	IF	1:250

Supplementary Figure 1 – Workflow for image analysis using the OrientationJ plugin for ImageJ/Fiji.

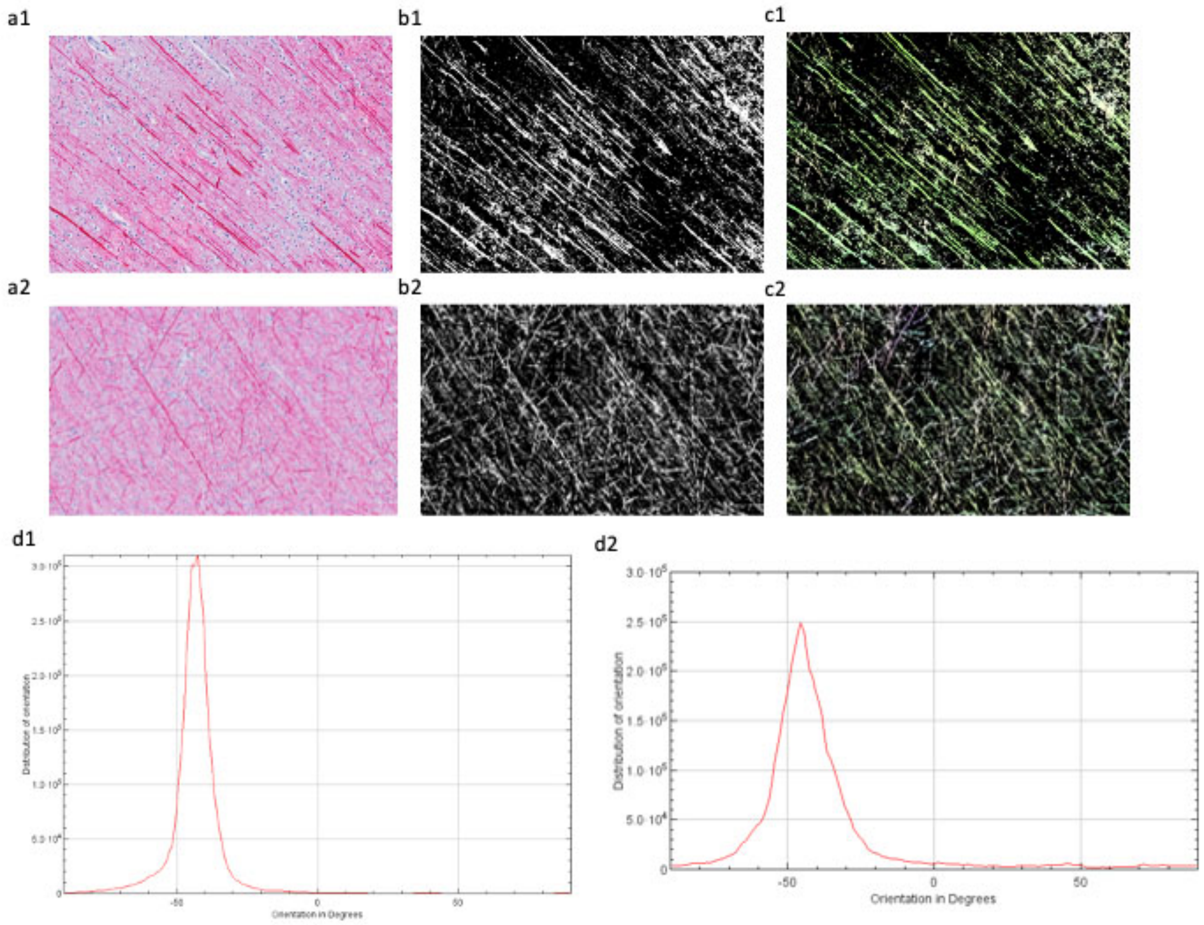

a1,2) Representative images of neurofilament light chain (NFL) immunopositive sections of the primary motor cortex.

b1,2) Results from colour deconvolution and thresholding (using the 'Default' method) to detect NFL immunopositive axons.

c1,2) Colour maps of NFL immunopositive axon orientation produced by OrientationJ, where hue represents orientation and saturation represents coherency. The size of the Gaussian-shaped window was estimated by calculating the average minimum Feret distance of the axons detected in b.

d) Histogram of NFL immunopositive axon orientation for a control (d1) and case of Parkinson's disease (d2) produced by OrientationJ. Coherency was obtained by the *Dominant Direction* function from OrientationJ

Supplementary Figure 2 – Representative total protein membranes used for normalisation/correction

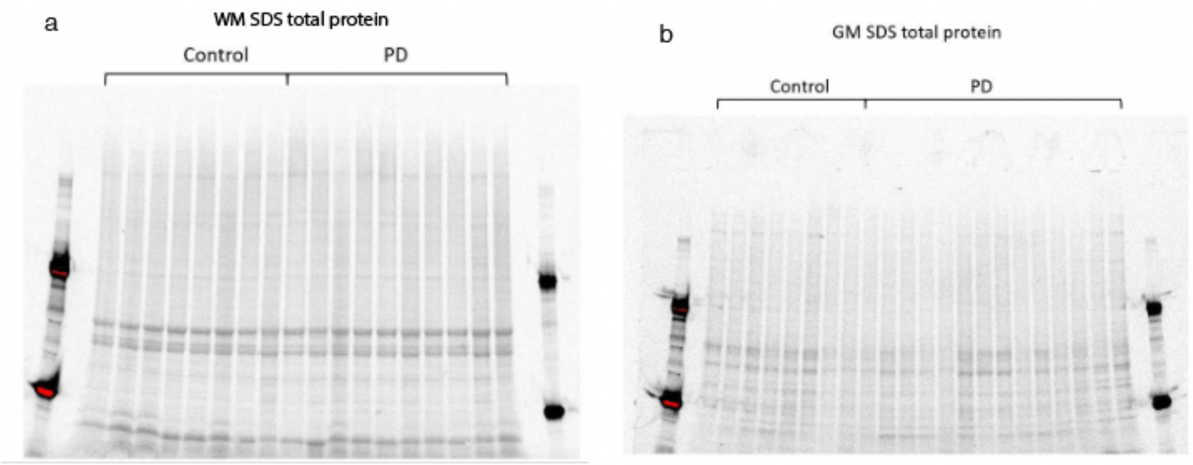

Insoluble proteins were serially extracted from fresh-frozen brain tissue of primary motor cortex and white matter using 5% sodium dodecyl sulfate (SDS) and high-speed centrifugation.

a) Insoluble SDS total protein Western blots in the white matter (WM) of controls and Parkinson's disease (PD) cases.

b) Insoluble SDS total protein Western blots in the grey matter (GM) of controls and Parkinson's disease (PD) cases.

Supplementary Figure 3 – Comparison of dopamine transporter PET/MRI between prodromal motor Parkinson's disease patients and healthy controls

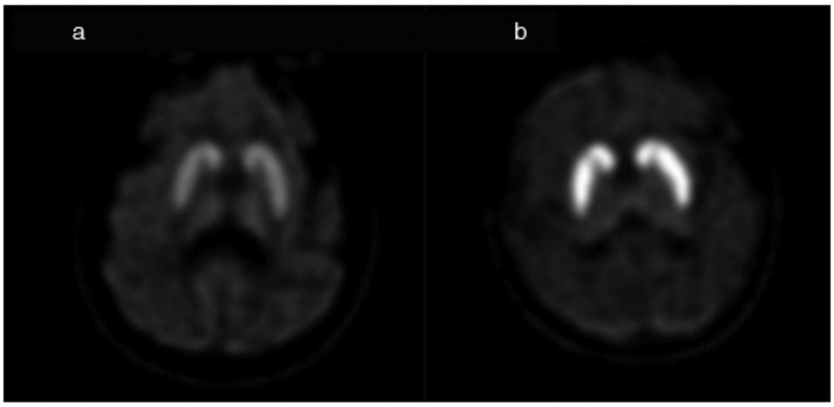

C)

	iRBD			HC		
	Left	Right	*p* value	Left	Right	*p* value
DAT SBR in the Caudate	1.45±0.24	1.46±0.28	0.71	2.94±0.64	3.07±0.86	1.00
DAT SBR in the Putamen	1.78±0.25	1.80±0.24	0.90	4.07±1.26	4.05±1.15	1.00

	Left	Right	*p* value
DAT SBR in the Caudate	51±8%	52±9%	0.71
DAT SBR in the Putamen	56±6%	56±6%	0.90

Basal ganglia dopamine transporter (DAT) was assessed using 18F-N-(3-fluoropropyl)-2β-carbon ethoxy-3β-(4-iodophenyl) nortropane (18F-FP-CIT) on a hybrid positron emission tomography (PET)/ magnetic resonance imaging (MRI) machine.

a) A representative patient with prodromal motor PD showing decreased dopamine transporter (DAT) binding in the striatum.

b) A representative control showing normal dopamine transporter (DAT) binding in the striatum

c) Data showing the dopamine transporter (DAT) specific binding ratio (SBR) between the left and right sides of the basal ganglia in controls and prodromal Parkinson's disease (iRBD) as well as the percentage of control DAT signal (mean ± SD) in prodromal PD from visit 2 (n=8). There was no difference between basal ganglia sides for those with prodromal PD (Mann-Whitney test *p* value given).

Supplementary Figure 4 – Other cytoskeleton proteins and their correlations with neurofilament triplet proteins in the white matter underlying the motor cortex

a

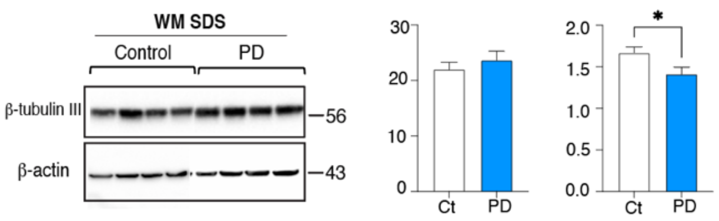

b

	SDS NFL	SDS NFM	SDS NFH	SDS β-tubulin III	SDS β-actin
SDS NFL	1	0.612	0.622	0.187	0.137
SDS NFM	0.612	1	0.54	0.096	0.115
SDS NFH	0.622	0.54	1	-0.067	0.199
SDS β-tubulin III	0.187	0.096	-0.067	1	0.432
SDS β-actin	0.137	0.115	0.199	0.432	1.000

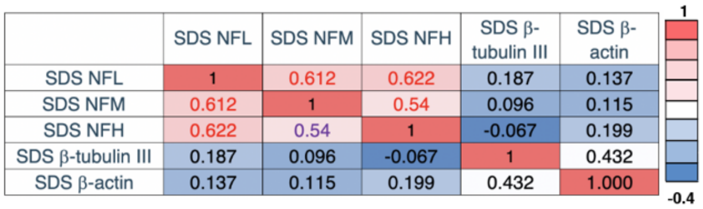

a) Representative western immunoblots of β-tubulin III and β-actin in the white matter of motor Parkinson's disease (PD, n = 8) and controls (n = 10). Column graphs represent mean levels ± standard error of the mean. Mann-Whitney test. * $p < 0.05$.

b) Spearman's rank correlations showed no correlations between the levels of β-tubulin III or β-actin with neurofilament light (NFL), medium (NFM or heavy chain (NFH). ρ values are colour coded as a heatmap. Font colour represents levels of *p* values: black- $p > 0.05$, purple- $p < 0.05$, red- $p < 0.01$.

Supplementary Figure 5 – Axonal pathology and myelination in the white matter underlying the motor and anterior cingulate cortex

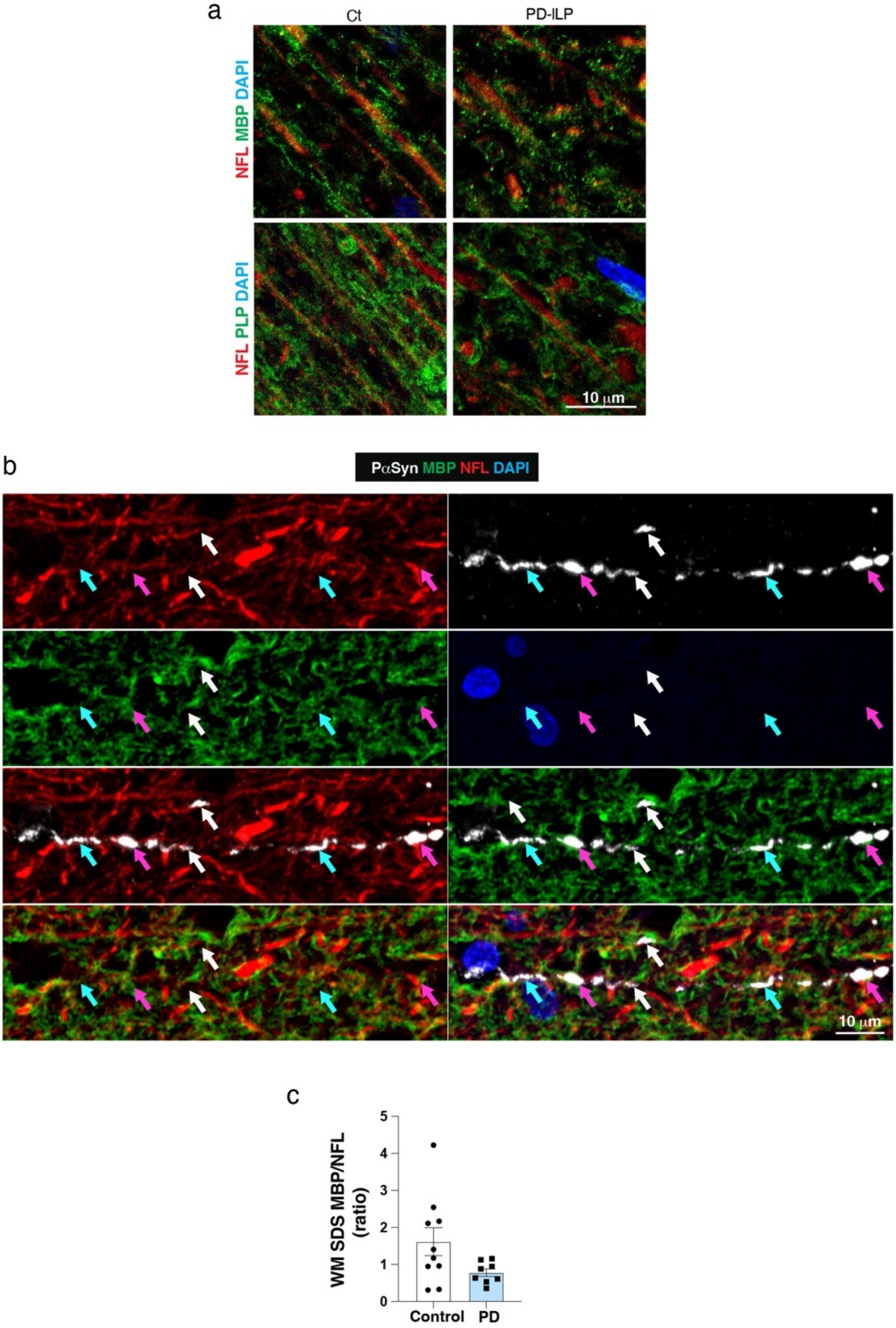

a) Stimulated emission depletion microscopy images of myelinated axons labelled with neurofilament light chain (NFL, red) and myelin basic protein (MBP, green) or myelin proteolipid protein (PLP, green) with glial

nuclei labelled with DAPI (blue) in the white matter underlying the motor cortex in a representative control (Ct) and patient with Parkinson's disease and late-stage Lewy pathology (PD-lLP).

b) In the white matter underlying the anterior cingulate cortex of patients with Parkinson's disease and late-stage Lewy pathology, Lewy pathology is more severe than in the white matter underlying the motor cortex in the same cases [9]. Lewy neurites (white signals) were often observed in segments in demyelinated axons (white arrows) or myelin demolishing axons (light blue arrows). Enlarged bean-like s129-αSyn aggregation were observed to mingle with unstructured myelin (pink arrows).

c) Scatter plot graph (mean with SEM) was presented for the ratio of protein levels between MBP and NFL (Mann-Whitney U test, p = 0.08).

Supplementary Figure 6 – Ratio of phosphorylated αSyn to pan αSyn in the motor cortex

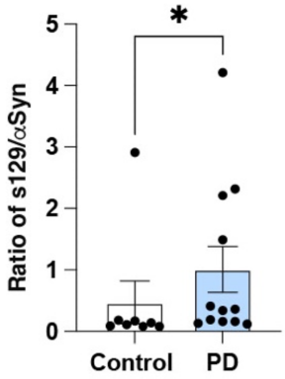

Scatter plot graph (mean ± standard error) of the ratio of protein levels between phosphorylated αSyn and pan αSyn (Mann-Whitney U test, * $p < 0.05$).

Supplementary Figure 7 – The size and ratio of oligodendrocytes in the motor cortex

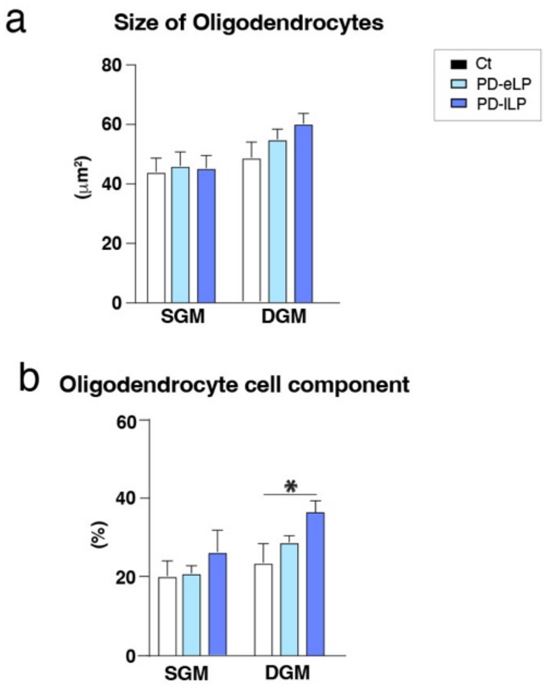

The size (a) and proportion (b) of tubulin polymerization promoting protein (TPPP) immunopositive oligodendrocytes in each group are shown as bar graphs (mean ± standard error, n = 8-13 per group). Statistical difference was tested with the Kruskal-Wallis (* $p < 0.05$) followed by Dunn's multiple comparisons ($p = 0.057$ between control and PD-late Lewy pathology). Ct, control; PD-eLP, PD with early Lewy pathology; PD-lLP, PD with late Lewy pathology; SGM, superficial cortical grey matter; DGM, deep cortical grey matter.
